# Effects of water temperature on freshwater macroinvertebrates: a systematic review

**DOI:** 10.1111/brv.12903

**Published:** 2022-09-29

**Authors:** Luca Bonacina, Federica Fasano, Valeria Mezzanotte, Riccardo Fornaroli

**Affiliations:** ^1^ Department of Earth and Environmental Sciences (DISAT) University of Milano‐Bicocca Piazza della Scienza 1 20126 Milan Italy

**Keywords:** aquatic insects, thermal conditions, inland waters, biotic response, climate change, conservation

## Abstract

Water temperature is one of the main abiotic factors affecting the structure and functioning of aquatic ecosystems and its alteration can have important effects on biological communities. Macroinvertebrates are excellent bio‐indicators and have been used for decades to assess the status of aquatic ecosystems as a result of environmental stresses; however, their responses to temperature are poorly documented and have not been systematically evaluated. The aims of this review are: (*i*) to collate and summarize responses of freshwater macroinvertebrates to different temperature conditions, comparing the results of experimental and theoretical studies; (*ii*) to understand how the focus of research on the effects of temperature on macroinvertebrates has changed during the last 51 years; and (*iii*) to identify research gaps regarding temperature responses, ecosystem types, organism groups, spatiotemporal scales, and geographical regions to suggest possible research directions. We performed a comparative assessment of 223 publications that specifically consider freshwater macroinvertebrates and address the effects of temperature. Short‐term studies performed in the laboratory and focusing on insects exposed to a range of temperatures dominated. Field studies were carried out mainly in Europe, at catchment scale and almost exclusively in rivers; they mainly investigated responses to water thermal regime at the community scale. The most frequent biological responses tested were growth rate, fecundity and the time and length of emergence, whereas ecological responses mainly involved composition, richness, and distribution. Thermal research on freshwater macroinvertebrates has undergone a shift since the 2000s when studies involving extended spatiotemporal scales and investigating the effects of global warming first appeared. In addition, recent studies have considered the effects of temperature at genetic and evolutionary scales. Our review revealed that the effects of temperature on macroinvertebrates are manifold with implications at different levels, from genes to communities. However, community‐level physiological, phenological and fitness responses tested on individuals or populations should be studied in more detail given their macroecological effects are likely to be enhanced by climate warming. In addition, most field studies at regional scales have used air temperature as a proxy for water temperature; obtaining accurate water temperature data in future studies will be important to allow proper consideration of the spatial thermal heterogeneity of water bodies and any effects on macroinvertebrate distribution patterns. Finally, we found an uneven number of studies across different ecosystems and geographic areas, with lentic bodies and regions outside the West underrepresented. It will also be crucial to include macroinvertebrates of high‐altitude and tropical areas in future work because these groups are most vulnerable to climate warming for multiple reasons. Further studies on temperature–macroinvertebrate relationships are needed to fill the current gaps and facilitate appropriate conservation strategies for freshwater ecosystems in an anthropogenic‐driven era.

## I. INTRODUCTION

### (1) Macroinvertebrates

Aquatic macroinvertebrates are a heterogeneous group, consisting of aquatic invertebrates bigger than 500 μm (Hauer & Resh, 2017). They are found in marine and freshwater ecosystems, including seas, rivers, streams, springs, lakes, ponds, lagoons, wetlands, and transitional ecosystems. Macroinvertebrate communities are diverse and include thousands of species belonging to phyla such as Arthropoda [Crustacea (Amphipoda and Isopoda) and Insecta (Coleoptera, Diptera, Heteroptera, Odonata, Neuroptera, Ephemeroptera, Plecoptera and Trichoptera)], Mollusca (Gastropoda and Bivalvia), Annelida, Nematoda, Platyhelminthes, Porifera, Cnidaria and Bryozoa (Konrad, Brasher & May, 2008; Resh, 2008; Astorga *et al*., 2011; Demars *et al*., 2012). This review focuses only on freshwater macroinvertebrates.

Macroinvertebrates play important trophic roles in aquatic communities as a major component of secondary producers; they have a key role in food webs, linking organic matter resources with upper trophic levels (Merritt, Cummins & Berg, 2017). Macroinvertebrates are a highly diversified group of organisms adapted to live in wide‐ranging hydrological and trophic conditions. This ubiquity reflects their evolutionary histories (Will & Resh, 2008), including a wide variety of reproductive, phenological, trophic, metabolic, physiological and behavioural strategies adapted to their specific environments (Hauer & Resh, 2017). For example, some species utilize dormant eggs to survive dry conditions, or a diapause period when environmental conditions are harsh (Tougeron, 2019). Their life cycles range from multivoltine to semivoltine depending on the taxon. Populations of some species can switch to different life‐cycle strategies depending on their geographical distribution and climatic conditions (Lamberti *et al*., 1987; Braune *et al*., 2008; Everall *et al*., 2015). Behavioural adaptations include differentiation of the ability to migrate and colonize new habitats to search for food sources or to avoid predators. According to the River Continuum Concept, in lotic ecosystems the trophic composition of the macroinvertebrate community changes along a watercourse due to gradually changing environmental conditions and resource availability. Allochthonous organic inputs decrease from upstream to downstream while autochthonous primary production increases. As a consequence, the partitioning of collectors/shredders/grazers and predators changes, as does the ratio of gross primary productivity and community respiration (Vannote *et al*., 1980).

For all these reasons, the study of macroinvertebrates has been (Hynes, 1970; Cummins, 1974; Allan & Castillo, 2007) and will continue to be (Moore & Schindler, 2008; Sundermann *et al*., 2011; Giersch *et al*., 2015; Cañedo‐Argüelles *et al*., 2020) a central part of aquatic ecology.

Macroinvertebrates are widely used as bioindicators (Holt & Miller, 2011) because they are common and abundant, well studied and provide measurable responses to environmental stress. An unimpaired freshwater body commonly contains dozens of taxa, representing a wide range of habitat preferences and life‐history strategies. This taxonomic and functional diversity can reflect responses to multiple environmental conditions, stressors, and disturbances, including the presence of fine sediment, metals, nutrients, invasive species, and hydrologic alterations. Accordingly, benthic invertebrates have been increasingly used as bioindicators since the 1950s (Beck, 1955), and many ecological indices based on macroinvertebrate assemblages have been developed. Such indices have been used to evaluate the effects of temperature on features of macroinvertebrate communities, for example, total taxa richness, relative proportion and/or richness of Ephemeroptera, Plecoptera and Trichoptera (EPT) (Jourdan *et al*., 2018; Fornaroli *et al*., 2020; Krajenbrink *et al*., 2021), Simpson and Shannon diversity indices (Arai *et al*., 2015) and Jaccard and Bray–Curtis indices of similarity (Burgmer, Hillebrand & Pfenninger, 2007).

### (2) The role of temperature

Water temperature is one of the primary factors affecting macroinvertebrates. Recording how benthic invertebrates respond to changes in water temperature is crucial to understanding the effects of climate change on freshwater ecosystems (Jourdan *et al*., 2018). Moreover, a deeper knowledge of temperature–biology relationships may allow researchers to disentangle the interacting effects of other aquatic ecosystem stressors like pollution, flow alteration and habitat reduction.

**Fig. 1:**
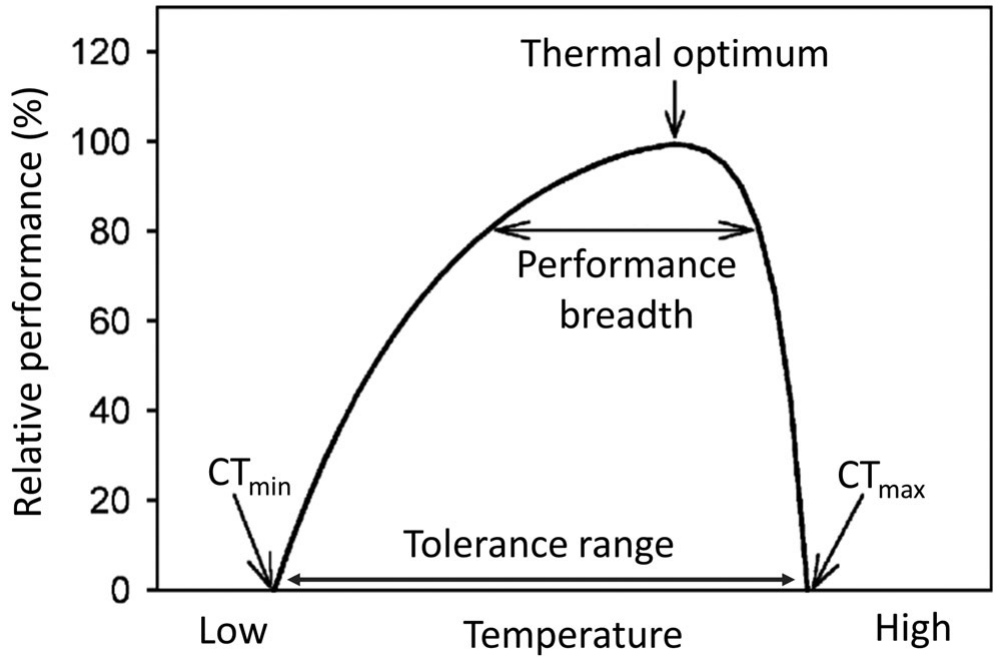
Typical thermal performance curve for ectotherms (adapted from Huey & Stevenson, 1979). CT_max_, critical thermal maximum; CT_min_, critical thermal minimum.

Water temperature influences the solubility of gases (e.g. oxygen) and pollutants, toxicity of chemicals, pH, density, and electrical conductivity. Moreover, temperature controls nutrient cycles, organic matter degradation and primary production. Generally, higher temperatures promote microbial metabolic activity and photosynthesis and affect the development and performance of biotic communities. Each species requires a specific temperature range for optimal performance. The performance–temperature curve is an asymmetric bell curve where performance is maximized at an optimal body temperature and the extremes represent the critical thermal limits [minimum (CT_min_) and maximum (CT_max_); Fig. 1; see Table 1 for glossary]. The range of body temperatures over which performance is equal to or greater than a specified level is called the thermal performance breadth and indicates the width of the individual thermal niche (Angilletta *et al*., 2002). Stenothermal macroinvertebrate species occupy a restricted temperature range while eurythermal species can tolerate a wider one (Jones, Muhlfeld & Haner, 2017). For many organisms, temperature changes can trigger specific life‐cycle phases such as migration, embryonic and larval development, egg hatching, and timing and duration of emergence (Angilletta, 2009*a*,*b*). When approaching their thermal limits, organisms show signs of stress, resulting in changes in behaviour (migration, drift and locomotion) (Sherberger *et al*., 1977; Bruno *et al*., 2012), physiology and metabolism (respiration, assimilation and excretion, growth rate and body size) (Sweeney, 1978; Zimmerman & Wissing, 1978), reproductive strategies (fecundity, hatching time and success) (Brittain & Mutch, 1984; Everall *et al*., 2015), and susceptibility to predators (Smolinský & Gvoždík, 2014; Śniegula, Golab & Johansson, 2019), pathogens and parasites (Pritchard & Zloty, 1994). Other responses caused by temperature alterations can affect species distribution and macroinvertebrate community structure through invasions of alien species as well as extinction of vulnerable ones (Dallas & Rivers‐Moore, 2014). The effects of temperature changes are often cumulative and also can vary depending on developmental stage (Dallas & Ross‐Gillespie, 2015).

**Table 1:** Glossary.

Climate warming	Long‐term increase of average air temperature near the surface of Earth also involving increased water temperature. This warming trend has been underway for a long time but has increased significantly in recent decades due to human activities.
Ectotherms	Organisms for which habitat temperatures determine body temperatures. Ectotherms may have a variable body temperature or may maintain a stable body temperature by moving from one site to another. Ectotherms are unable to produce and conserve adequate metabolic heat to maintain a body temperature above the external temperature. Antonym of endotherms.
Eurythermal species	Species tolerating a wide temperature range. Antonym of stenothermal species.
Hemimetabolous insects	Insects that undergo incomplete or partial metamorphosis (e.g. Plecoptera, Ephemeroptera, Odonata).
Holometabolous insects	Insects that undergo complete metamorphosis (e.g. Trichoptera, Diptera, Coleoptera).
Stenothermal species	Species that can only live in a narrow range of temperatures. Antonym of eurythermal species.
Temperature changes	Refers to a generic change in the water temperature conditions.
	*Temperature alteration*: temperature change of a water body caused by anthropogenic causes (such as thermal effluents of nuclear power plants).
	*Temperature gradient*: water temperature variation over a specified distance. In field studies, it includes lake temperature stratification and the altitudinal gradient of a mountain stream or a geothermal watercourse. In experimental studies, it can be set in tanks that reproduce or manipulate the natural gradient.
	*Temperature range*: defined as the different temperature levels set in experimental studies to which organisms are exposed to assess the temperature dependence of life‐history traits.
	*Temperature variation*: defined as generic changes in experimental studies where water temperature is increased or decreased to simulate daily/seasonal fluctuations or temperature shocks.
	*Thermal shocks* are sudden and repeated changes in water temperature. *Thermopeaking* is thermal shock occurring in a watercourse impacted by hydropeaking.
	*Thermal stress*: organismal responses when the water temperature approaches species‐specific critical thermal limits.
Temperature conditions	The thermal environment in which organisms/populations/communities live. Herein we classify temperature conditions for experimental studies as: high temperature, temperature variation, or temperature range; and in field studies as: climate warming, temperature regime, or constant temperature regime.
Thermal divergence	Refers to the presence of populations of the same species that show a different performance with respect to temperature due to their different evolutionary histories.
Temperature effects	Biological and ecological outcomes of temperature. They concern organism physiology, metabolism, phenology, fitness, behaviour, community ecology and evolution.
Temperature regime	Water temperature variability of a waterbody on both the temporal (e.g. daily, seasonal) and spatial scales.
	*Natural temperature regime*: spatial and temporal water temperature variability of a waterbody that is not affected by direct anthropogenic impacts.
	*Constant temperature regime*: the temperature regime of a waterbody characterized by constant temperature (typically karst streams and springs).
Thermal niche	The range of temperatures allowing population growth, or the temperature requirements of a species within its ecosystem. Depending on the methods used for its estimation the thermal niche is evaluated from organism traits (based on measurements of temperature dependence for life‐history or other traits) and from biogeographical indications (based on the climatic region of the species distribution).
Thermal performance breadth	Range of body temperatures over which performance is greater than or equal to an arbitrary level of performance, usually expressed as a percentage of the maximal level. For example, an 80% performance breadth is the range of body temperatures over which performance is greater than or equal to 80% of the maximum (Fig. 1).
	*Critical thermal maximum (CT*_*max*_ *) and minimum (CT*_*min*_ *)*: maximum and minimum temperatures allowing normal performance.
	*Thermal optimum*: temperature corresponding to organismal maximum performance.
	*Thermal tolerance*: the difference between CT_max_ and CT_min_.
Voltinism	The frequency or number of annual broods of an organism within a year.

Due to the strong link between temperature, life cycle and development of macroinvertebrates, several hypotheses have been proposed to explain how macroinvertebrates respond to different temperature conditions. The first attempt to establish a relationship between environmental temperature and biological mechanisms was the thermal equilibrium hypothesis (TEH) (Sweeney & Vannote, 1978). This hypothesis proposed that: (*i*) for each species, maximum adult body size reflects an equilibrium among developmental processes regulated by temperature, including larval growth rate and duration, maturation period of adult structures and rate of maturation processes, with maximum adult size and fecundity achieved at an optimum temperature within the thermal tolerance range; (*ii*) both locally and over large geographic areas, a species distribution is limited by fecundity and adult size, which gradually diminish with increasingly cold or warm temperature cycles (Sweeney, 1978). It follows that small adult size and reduced fecundity (leading to low levels of population recruitment and growth) are the basis by which temperature changes cause the extinction of aquatic populations (Sweeney *et al*., 2018). Other theories regarding the relationship between temperature, biological mechanisms and ecological patterns have been proposed (Atkinson, 1994; Kingsolver & Huey, 2008). During the last 20 years, researchers have begun to investigate the effects of global warming on aquatic communities. Due to the rapid development of this research and the increasing number of studies assessing the effects of temperature changes on freshwater communities, we carried out a review of the scientific literature published in the last 50 years to: (*i*) provide an updated, and comprehensive review of responses of freshwater macroinvertebrates to water temperature; (*ii*) understand how the focus of research on the effects of temperature on macroinvertebrates has changed during the last 50 years; and (*iii*) identify current research gaps regarding ecosystem types, taxa, spatial and temporal scales, and climatic regions to suggest future research directions.

## II. METHODOLOGY

We performed a search for relevant publications in the ISI *Web of Science* (WoS) database using the search string: Title = ((“temperature”) AND (“macroinvertebrates”) AND (“biotic response”) NOT (“other”)), where: “temperature” includes: temperature* OR thermal OR warm* OR heat OR climate change OR thermic; “macroinvertebrates” includes: invertebrat* OR macroinvertebrat* OR (aquatic AND insect*) OR benthos OR benthic OR Plecoptera OR Ephemeroptera OR Trichoptera OR (aquatic AND Coleoptera) OR (aquatic AND Diptera) OR mayfl* OR stonefl* OR caddisfl* OR (aquatic AND fly) OR (aquatic AND beetl*) OR chironomid* OR freshwater biota OR Odonata OR damselfl* OR neuropteran* OR Neuroptera OR Megaloptera OR megalopteran* OR dragonfl* OR (aquatic AND Heteroptera) OR (aquatic AND Hemiptera*) OR midg*; “biotic response” includes: stress* OR variation* OR dynamic* OR dietary OR food OR effect OR nich* OR phenology OR (life AND cycle) OR trait* OR growth OR reproduction OR mortality OR diseas* OR behaviour* OR performance OR dimension* OR size OR fitness OR success OR voltinism OR flexibility OR emergence OR (egg AND development) OR richness OR composition OR drift OR migration OR spatial OR pattern* OR gene* OR feeding OR predation; and “other” includes: fish* OR marine OR sea* OR ocean* OR coast* OR plankton OR brackish OR meiofauna OR oyster OR terrestrial OR soil OR coral OR foraminifer* OR alga OR diatom*.

The literature search considered both original research and review papers published between January 1970 and December 2020 and generated 425 records. After examining the abstracts, only papers related to freshwater macroinvertebrates were retained while papers dealing with marine, lagoon or estuary ecosystems and vertebrates or micro‐invertebrates were excluded. Ecotoxicological articles were also excluded when temperature was not the main focus so the number of publications dropped to 269. Finally, each paper was read in full to confirm its relevance to our review and 223 publications were retained (Fig. S1). For each research article (*N* = 218) in our final database we recorded information including the ecological unit investigated (community, population, gene), focal taxon (recorded to order), temperature conditions, spatiotemporal scale of the study and sampling frequency (see Table 2 for full list of categories). Biotic responses were collated into six categories of effect (physiological and metabolic, phenological, fitness, behavioural, ecological, evolutionary; Table 3). For each category listed in Tables 2 and 3, multiple selections were possible for a single paper, i.e. if different temperature conditions were investigated by a single publication, thus the total number of studies differs between categories and can exceed the number of publications (Table S1). To provide a comprehensive overview of the selected papers we carried out several analyses. (*i*) We performed a distribution analysis for each category, with the results presented in cumulative bar plots reporting the relative percentages of studies across ecosystem type, spatial and temporal scale, survey frequency, level of investigation and ecological unit as well as temperature conditions and other stresses investigated. We used pie‐donuts charts to summarize the studied taxonomic groups and the types of effects and responses investigated. We performed principal components analyses (PCAs) to investigate the patterns of reported responses associated with ecosystem, temperature conditions, spatial scale, level of investigation, organism and continent (Stendera *et al*., 2012). (*ii*) To assess how the focus of thermal research has changed during the last 51 years, we plotted bubble grid charts for each subcategory of temporal scale, spatial scale, ecological unit, and temperature conditions. (*iii*) We used the above results and a global map showing the number of studies from each country/climatic region to identify areas where thermal studies on freshwater macroinvertebrates are still needed. All statistical analyses were performed using R project software (www.R-project.org) except for pie‐donuts charts that were elaborated using Python (www.python.org) and the map that was drawn in Qgis (www.qgis.org).

**Table 2:** Categories and sub‐categories of information drawn from the selected publications.

Category	Description	Sub‐categories
Ecosystem	Type of freshwater ecosystem considered	Lotic ecosystems: river, channel, spring
		Lentic ecosystems: lake, pond
		Laboratory^a^
Study	Type of study	Experimental, theoretical
Continent	Continent where the study was performed	Africa, Antarctica, Asia, Europe, Oceania, North America, Central America, South America
Climatic region	Climate according to the Köppen classification	Tropical, arid, temperate, cold, polar
Spatial scale	Environmental scale of the monitoring	Site‐specific, catchment, regional, ecoregion, continental, global
Temporal scale	Period of the study	Decades (<100 years), years (<10 years), months (<1 year), days (<1 month), punctual
Survey frequency	Frequency of sampling/observations in the field or laboratory	Annual, seasonal, monthly, weekly, daily, hourly, subhourly, punctual
Level of investigation	Taxonomic level of the investigation	Order, family, genus, species
Ecological unit	Investigated level of biological organization	Community, population,^b^ gene
Organism	Type of organism investigated	Taxonomic order
Temperature conditions	Category of temperature changes	Laboratory: high temperature, temperature variation, temperature range
		Field: climate warming, temperature regime, constant temperature regime
Other stress	Other stresses/factors investigated	Predation, pollution, photoperiod, oxygen availability, nutrient concentration, humidity, habitat, food availability, flow, other.

^a^ Includes water‐filled containers and baths, temperature‐gradient tanks, flow‐through systems, microcosms and mesocosms.

^b^ Studies not referred to community or gene levels were attributed to population as individual responses were always investigated as representative of the population.

**Table 3:** List of the biotic responses measured in relation to temperature in the selected publications.

Effects	Biotic responses
Physiological and metabolic	Gene expression, osmoregulation ability, respiration, body size and growth rate, size at emergence, assimilation/excretion, thermal limits
Phenological	Total time of development, time and length of hatching, time and length of emergence, voltinism, colour
Fitness	Fecundity and hatching success, larval recruitment
Behavioural	Drift, migration, predation, feeding
Ecological	Richness, taxonomic composition, density, distribution, food‐chain length, community structure and trophic role, secondary production
Evolutionary	Genetic diversity

## III. RESULTS

### (1) General overview

**Fig. 2:**
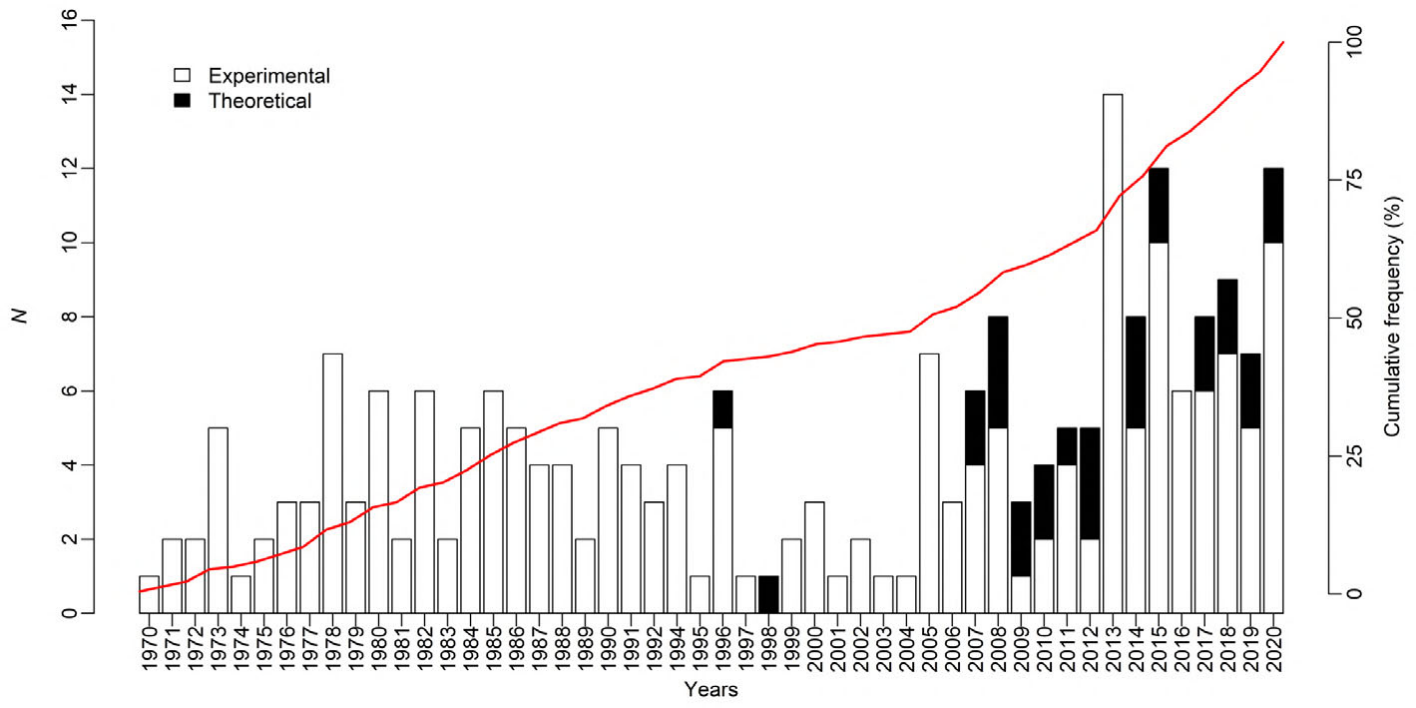
Number of publications (*N* = 223) on aquatic macroinvertebrate responses to temperature per year from 1970 to 2020.

Our literature search identified 223 relevant publications [218 research articles and 5 reviews (Fig. 2; Tables 4 and S1)]. During the last decade, the number of publications reporting macroinvertebrate responses to temperature has increased, with a notable upturn in the number of theoretical studies.

**Table 4:** Responses of macroinvertebrates to temperature summarized from the studies included in our database. Additional references not included in the database are identified by an asterisk (*).

Effect	Biotic response	Overall patterns	References
Physiological and metabolic	Gene expression	Temperature influences the expression of genes involved in thermal regulation and their speed of replication.	Chou *et al*. (2018); * Ebner *et al*. (2019); Hotaling *et al*. (2020); Karouna‐Renier & Zehr (1999); Kim *et al*. (2017); Lencioni *et al*. (2013); Lopez‐Martinez *et al*. (2008); Martín‐Folgar *et al*. (2015); * Schmeller *et al*. (2018); Swaegers *et al*. (2020); Teets *et al*. (2013)
	Osmoregulation ability	Higher temperatures imply higher osmotic concentration of haemolymph in aquatic arthropods.	Colburn (1983); * Orr & Buchwalter (2020)
	Respiration	Respiration rates increase with temperature.	* Bergström *et al*. (2010); Burton *et al*. (1976); Forster *et al*. (2012); Hamburger *et al*. (1994); Howell & Voshell (1982); Jones *et al*. (2018); Kim *et al*. (2017); Modlin & Jayne (1981); Rotvit & Jacobsen (2013); * Sinsabaugh (1997); Sweeney (1978)
	Body size and growth rate	Growth rate increases with warming until an optimum temperature, beyond which it declines. Females may invest resources into fecundity at the expense of the growth of somatic tissues, so females may be smaller than males in warm conditions.	Abbott (2013); * Atkinson (1994, 1995); * Bergmann (1847); Bottová *et al*. (2013*a*,*b*); Brittain (1983); Brittain *et al*. (1984); Brittain & Mutch (1984); Chadwick & Feminella (2001); Chavez *et al*. (2015); Cogo *et al*. (2020); Corkum & Hanes (1992); Culler *et al*. (2014); Elliott (1987); Fahy (1973); Fenoglio *et al*. (2005); * Forster *et al*. (2012); Frouz *et al*. (2002); Fuller & Fry (1991); Giberson & Rosenberg (1992*a*); Gresens (1997); Hamburger *et al*. (1994); Hassall (2013); Hauer & Benke (1991); Hayashi (1988, 1996); Hines *et al*. (2016); * Horne *et al*. (2015, 2018); Howell & Voshell (1982); Humpesch (1981); Huryn (1996); Imholt *et al*. (2010); Ingram (1976); * Kingsolver & Huey (2008); Krishnaraj & Pritchard (1995); Leggott & Pritchard (1985); Li *et al*. (2009); Lillehammer (1985, 1986); Maier *et al*. (1990); Markarian (1980); Martins *et al*. (2016); McCafferty & Pereira (1984); McKie & Cranston (2005); McKie & Pearson (2006); McKie *et al*. (2004); Mochizuki *et al*. (2006); Moody *et al*. (2017); Muthukrishnan *et al*. (1988); Newell & Minshall (1978); Nilsson‐Örtman *et al*. (2013*a*,*b*, 2012, 2014); Ouahsine *et al*. (1996); Perry *et al*. (1987); Péry & Garric (2006); Pickup & Thompson (1990); Piggott *et al*. (2015); Pritchard & Pelchat (1977); Pritchard & Zloty (1994); Procter (1973); Rader & Ward (1990); Rempel & Carter (1987); Reynolds & Benke (2005); Rosillon (1988); Sarvala (1979); Scherr *et al*. (2010); Śniegula *et al*. (2019); Söderström (1988); Starr & McIntyre (2020); Stoks *et al*. (2012); Storey (1987); Suhling *et al*. (2015); Šupina *et al*. (2020); Sweeney (1978); Sweeney & Vannote (1978, 1984, 1986); Sweeney *et al*. (1986*a*,*b*); Turner & Williams (2005); Tüzün *et al*. (2017); Van Doorslaer & Stoks (2005*a*,*b*); * Verberk *et al*. (2021); Vogt *et al*. (2007); Wagner (1990, 2005); Wright *et al*. (1982); Zimmerman & Wissing (1978)
	Size at emergence	High temperatures lead to faster growth and smaller emergence size due to accelerated metabolism. Low temperatures slow down the growth rate potentially leading to larger emergence size. Temperature can promote sexual size dimorphism.	Abbott (2013); Brittain (1983); Chacón *et al*. (2016); Farkas *et al*. (2013); Giberson & Rosenberg (1992*a*); Hayashi (1988, 1996); Jonsson *et al*. (2015); Langford (1975); McCauley *et al*. (2015, 2018); Rosillon (1988); Śniegula *et al*. (2019); Söderström (1988); Sweeney & Vannote (1978, 1986); Sweeney *et al*. (1986*b*); Turner & Williams (2005); Wonglersak *et al*. (2020)
	Assimilation/ excretion	Higher temperatures enhance ingestion and excretion rates.	Anderson *et al*. (2017); Beracko & Revajová (2019); Bottová *et al*. (2013*a*,*b*); Culler *et al*. (2014); Martins *et al*. (2020); Moody *et al*. (2017); Muthukrishnan *et al*. (1988); Pandian *et al*. (1979); Péry & Garric (2006); Pickup & Thompson (1990); Stoks *et al*. (2012); Thompson (1978); Van Doorslaer & Stoks (2005*a*); Winterbourn *et al*. (2008); Zimmerman & Wissing (1978)
	Thermal limits	Stenothermal invertebrates occupy a small temperature range while eurytherms occupy wider ranges. Tropical species have narrower thermal tolerances compared to temperate ones.	* Brett (1952); Burton *et al*. (1976); Chadwick & Feminella (2001); Chou *et al*. (2018); Collier & Smith (2000); Cox & Rutherford (2000); * Dallas & Ketley (2011); * Dallas & Rivers‐Moore (2012); Danks (1978); Dickson & Walker (2015); Elliott (1987); Giberson & Rosenberg (1992*a*); Hotaling *et al*. (2020); Martin *et al*. (1976); McKie & Pearson (2006); McKie *et al*. (2004); Mochizuki *et al*. (2006); * Niedrist & Füreder (2020); * Polato *et al*. (2018); Rogowski & Stewart (2016); Rosillon (1988); Sawchyn & Church (1973); * Shah *et al*. (2017); Sherberger *et al*. (1977); Shoup & Houghton (2013); * Stewart *et al*. (2013); Suhling *et al*. (2015); Sweeney & Vannote (1986); Sweeney *et al*. (1986*a*,*b*); Vogt *et al*. (2007); Wellborn & Robinson (1996)
Phenological	Total time of development	High temperatures shorten the development time, leading to more rapid emergence.	Abbott (2013); Abdullahi & Laybourn‐Parry (1985); Bayoh & Lindsay (2003); Beracko & Revajová (2019); Elliott (1987); Fahy (1973); Frouz *et al*. (2002); Hauer & Benke (1991); Humpesch (1981); Huryn (1996); Imholt *et al*. (2010); Li *et al*. (2009); Mackay (1984); Maier *et al*. (1990); Marten (1990); McCafferty & Pereira (1984); McCauley *et al*. (2015); McKie & Pearson (2006); McKie *et al*. (2004); Pritchard & Pelchat (1977); Pritchard & Zloty (1994); Rosillon (1988); Śniegula *et al*. (2019); Söderström (1988); Sweeney & Vannote (1984); Sweeney *et al*. (1986*b*); Trottier (1971); Wagner (1990); Wright *et al*. (1982)
	Time and length of hatching	Hatching time decreases at higher temperatures. Low temperatures lengthen the hatching period and induce diapause.	Abdullahi & Laybourn‐Parry (1985); Bohle (1972); Bouton *et al*. (2011); Brittain (1977, 1991); Brittain & Campbell (1991); Brittain & Mutch (1984); Brittain *et al*. (1984); Corkum & Hanes (1992); Elliott (1972, 1978, 1986, 1987); Friesen *et al*. (1979); Frouz *et al*. (2002); Giberson & Rosenberg (1992*b*); Gillooly & Dodson (2000*a*); Gong *et al*. (2002); Humpesch & Elliott (1980); Humpesch (1980*a*,*b*, 1982); Ichikawa *et al*. (2017); Leggott & Pritchard (1985); Lillehammer ((1985, 1986)); Maier *et al*. (1990); Marten (1990); Mendonça *et al*. (2018); Muthukrishnan *et al*. (1988); Perry *et al*. (1987); Pritchard & Zloty (1994); Pritchard *et al*. (1996); Ross‐Gillespie *et al*. (2018); Sarvala (1979); Sawchyn & Church (1973); Strange (1985); Sweeney & Vannote (1984); Zwick (1996)
	Time and length of emergence	Increasing temperatures lead to earlier emergence.	Abdullahi & Laybourn‐Parry (1985); Chaćon *et al*. (2016); Cheney *et al*. (2019); Čmrlec *et al*. (2013); Coler & Kondratieff (1989); Danks (1978); Dickson & Walker (2015); Dingemanse & Kalkman (2008); Elliott (1987); Everall *et al*. (2015); Frouz *et al*. (2002); Hassall *et al*. (2007); Hayashi (1988, 1996); Huryn (1996); Imholt *et al*. (2010); Ingram (1976); Ivković *et al*. (2013); Jonsson *et al*. (2015); Killian & Lutz (1985); Langford (1975); Leggott & Pritchard (1985); Li *et al*. (2009); Lutz (1974); Maier *et al*. (1990); McCafferty & Pereira (1984); McCauley *et al*. (2015, 2018); McKie & Pearson (2006); Nebeker (1971); Perry *et al*. (1987); Péry & Garric (2006); Piggott *et al*. (2015); Pritchard & Zloty (1994); Procter (1973); Rempel & Carter (1987); Richter *et al*. (2008); Starr & McIntyre (2020); Šupina *et al*. (2020); Sweeney (1978); Sweeney & Vannote (1986); Sweeney *et al*. (1986*a*,*b*); Trottier (1971, 1973*a*,*b*); Villalobos‐Jimenez & Hassall (2017); Vogt *et al*. (2007); Watanabe *et al*. (1999); Wright *et al*. (1982)
	Voltinism	Higher temperatures favour a flexible life cycle and increase voltinism. Low temperatures cause longer developmental time and favour a univoltine cycle.	Beracko & Revajová (2019); Bottová *et al*. (2013*a*,*b*); Braune *et al*. (2008); Elliott (1987); Everall *et al*. (2015); Farkas *et al*. (2013); Mackay (1984); Newell & Minshall (1978); Pritchard & Zloty (1994); * Rivers‐Moore *et al*. (2012); Söndgerath *et al*. (2012)
	Colour	Temperature may interfere with colour regulation.	Abbott (2013); Bouton *et al*. (2011); Hayashi (1988); McCafferty & Pereira (1984)
Fitness	Fecundity and hatching success	Fitness is maximized at the optimal temperature. Elevated temperatures imply lower fecundity and faster hatching accompanied by a lower hatching success. Low temperatures promote large broods and higher fecundity in females.	Bayoh & Lindsay (2003); Bovill *et al*. (2019); Brittain (1977, 1991); Brittain & Campbell (1991); Brittain *et al*. (1984); Corkum & Hanes (1992); Elliott (1972, 1987, 1986); Friesen *et al*. (1979); Giberson & Rosenberg (1992*a*,*b*); Gillooly & Dodson (2000*a*); Gong *et al*. (2002); Humpesch & Elliott (1980); Humpesch (1982, 1981, 1980*a*,*b*); Ichikawa *et al*. (2017); Imholt *et al*. (2010); Leggott & Pritchard (1985); Lillehammer (1985, 1986); Marten (1990); McKie & Pearson (2006); Newell & Minshall (1978); Péry & Garric (2006); Pritchard & Zloty (1994); Rader & Ward (1990); Rempel & Carter (1987); Rosillon (1988); Ross‐Gillespie *et al*. (2018); Sarvala (1979); Sawchyn & Church (1973); Söderström (1988); Starr & McIntyre (2020); Strange (1985); Sweeney (1978); Sweeney & Vannote (1978, 1984); Sweeney *et al*. (1986*b*); Tennessen *et al*. (1982); Van Doorslaer & Stoks (2005*b*); Wright *et al*. (1982); Zwick (1996)
	Larval recruitment	Juvenile recruitment increases with temperature increase; at low temperatures hatching is inhibited.	Abbott (2013); Brittain (1991); Brittain *et al*. (1984); Chavez *et al*. (2015); Corkum & Hanes (1992); Danks (1978); Giberson & Rosenberg (1992*b*); Ingram (1976); Killian & Lutz (1985); Lencioni *et al*. (2013); Marten (1990); Martins *et al*. (2016); McCauley *et al*. (2015, 2018); Nilsson‐Örtman *et al*. (2014); Pritchard & Pelchat (1977); Śniegula *et al*. (2019); Storey (1987); Šupina *et al*. (2020); Tüzün *et al*. (2017); Van Doorslaer & Stoks (2005*b*); Wright *et al*. (1982); Zwick (1996)
Behavioural	Migration	Macroinvertebrates migrate to locate their preferred thermal environment	Shah *et al*. (2020); Sherberger *et al*. (1977); Trottier (1973*b*); Van Doorslaer & Stoks (2005*a*); * Waters (1965)
	Drift	Thermopeaking and high temperatures cause drift.	* Bruno *et al*. (2012); * Carolli *et al*. (2012); Coler & Kondratieff (1989); Dudgeon *et al*. (2020); Durrett & Pearson (1975); Piggott *et al*. (2015); Raddum (1985); Scherr *et al*. (2010); * Schülting *et al*. (2016); Wojtalik & Waters (1970)
	Predation	Predators may be more vulnerable to increasing temperature than their prey. Elevated temperatures compromise hunting capacity but also reduce avoidance ability in prey.	* Kishi *et al*. (2005); MacPhee *et al*. (2011); McKie & Pearson (2006); Quenta Herrera *et al*. (2018); * Rogowski & Stewart (2016); Sherberger *et al*. (1977); Smolinský & Gvoždík (2014); Śniegula *et al*. (2019)
	Feeding	At higher temperatures macroinvertebrates require more food/better food quality.	Bottová *et al*. (2013*a*); Gordon *et al*. (2018); Krishnaraj & Pritchard (1995); Navarro & Gonçalves Junior (2017); Pandian *et al*. (1979); Pickup & Thompson (1990); Śniegula *et al*. (2019)
Ecological	Richness	The number of species generally increases with increasing annual temperature ranges. The highest temperatures lead to an impoverished community with better survival of eurythermal and generalist species. Global warming facilitates the extinction of stenothermal species.	Arai *et al*. (2015); Arthur *et al*. (1982); Barquín & Death (2011); Besacier *et al*. (2019); Burgmer *et al*. (2007); * Castella *et al*. (2001); Chinnayakanahalli *et al*. (2011); Čmrlec *et al*. (2013); Contador *et al*. (2014); Conti *et al*. (2014); Dudgeon *et al*. (2020); Durance & Ormerod (2007, 2009); Eversham & Cooper (1998); Feuchtmayr *et al*. (2007); Floury *et al*. (2013); Glazier (2012); Gordon *et al*. (2018); Gustafson (2008); Haidekker & Hering (2008); Jackson *et al*. (2007); Jourdan *et al*. (2018); Munari (2011); Nyquist *et al*. (2020); Poff *et al*. (2010); Rasmussen (1982); Saltveit *et al*. (1994); Sandin *et al*. (2014); Voelz *et al*. (1994); * Ward & Stanford (1982); Wellborn & Robinson (1996); Worthington *et al*. (2015); Živić *et al*. (2014)
	Taxonomic composition	The macroinvertebrate community is generally more diversified in ecosystems characterized by wide daily and seasonal temperature variation. Global warming leads to homogenization of macroinvertebrate communities.	Arai *et al*. (2015); Arthur *et al*. (1982); Barquín & Death (2011); Besacier *et al*. (2019); Burgmer *et al*. (2007); Cerini *et al*. (2020); Chinnayakanahalli *et al*. (2011); Čmrlec *et al*. (2013); Coler & Kondratieff (1989); Contador *et al*. (2014); Conti *et al*. (2014); Cooper (1980); Dudgeon *et al*. (2020); Durance & Ormerod (2007, 2009); Feuchtmayr *et al*. (2007); Floury *et al*. (2013); * Fornaroli *et al*. (2020); Gustafson (2008); Haidekker & Hering (2008); Jackson *et al*. (2007); Jourdan *et al*. (2018); Munari (2011); Nyquist *et al*. (2020); Piggott *et al*. (2015); Poff *et al*. (2010); Rasmussen (1982); Saltveit *et al*. (1994); Sandin *et al*. (2014); Voelz *et al*. (1994); Worthington *et al*. (2015); Živić *et al*. (2014)
	Density	Temperature changes lead to alterations in species density depending on each species' thermal niche. Global warming increases the abundance of generalist species at the expenses of the stenothermal ones.	Arai *et al*. (2015); Arthur *et al*. (1982); Barquín & Death (2011); Besacier *et al*. (2019); Burgmer *et al*. (2007); Cheney *et al*. (2019); Čmrlec *et al*. (2013); Coler & Kondratieff (1989); Contador *et al*. (2014); Conti *et al*. (2014); Cooper (1980); Dudgeon *et al*. (2020); Durance & Ormerod (2007, 2009); Durrett & Pearson (1975); Feuchtmayr *et al*. (2007); Floury *et al*. (2013); Giberson & Rosenberg (1992*a*); Gustafson (2008); Haidekker & Hering (2008); Jackson *et al*. (2007); Jourdan *et al*. (2018); Nyquist *et al*. (2020); Poff *et al*. (2010); Raddum (1985); Rader & Ward (1990); Rasmussen (1982); Sandin *et al*. (2014); Voelz *et al*. (1994); Wagner (2005); Wellborn & Robinson (1996); Winterbourn *et al*. (2008); Worthington *et al*. (2015); Živić *et al*. (2014)
	Distribution	Distribution is influenced by a species' thermal optimum. Temperature changes affect the ecological niche occupied by the species promoting shifts in their distribution. Increasing temperatures cause the upstream spread of eurythermal species and reduce the habitat available for stenothermal species.	Arai *et al*. (2015); Baker & Feltmate (1989); Besacier *et al*. (2019); Cerini *et al*. (2020); Cheney *et al*. (2019); Chessman (2012); Chinnayakanahalli *et al*. (2011); Čmrlec *et al*. (2013); Conti *et al*. (2014); Cooper (1980); * Domish *et al*. (2011); Durance & Ormerod (2007); Eversham & Cooper (1998); Fenoglio *et al*. (2010); Haidekker & Hering (2008); Hering *et al*. (2009); * Mustonen *et al*. (2018); Nilsson‐Örtman *et al*. (2012, 2013*b*); Nyquist *et al*. (2020); Pires *et al*. (2018); Poff *et al*. (2010); Saltveit *et al*. (1994); Sandin *et al*. (2014); Silva *et al*. (2019); Söndgerath *et al*. (2012); Timoner *et al*. (2020); Winterbourn *et al*. (2008)
	Food‐chain length	Elevated temperatures cause an abrupt decline in food‐chain length); below a critical threshold the relationship between food‐chain length and temperature is not linear.	* Arim *et al*. (2007*b*); Glazier (2012)
	Community structure and trophic role	Temperature alterations may lead to changes in the composition of functional feeding groups. Grazers and scrapers appear especially vulnerable to warming.	Jonsson *et al*. (2015); Jourdan *et al*. (2018); * Pyne & Poff (2017); Sandin *et al*. (2014); Živić *et al*. (2014)
	Secondary production	Secondary production does not depend directly on temperature, although temperature can impact resource supply with effects on secondary production.	Bottová *et al*. (2013*a*); Ferreira *et al*. (2015); Humpesch (1981); * Junker *et al*. (2020); Newell & Minshall (1978); Patrick *et al*. (2019); Perry *et al*. (1987); Rader & Ward (1990); Sweeney & Vannote (1986)
Evolutionary	Genetic diversity	Temperature acts at an evolutionary scale causing thermal divergence in populations, promoting genetic divergence or causing fragmentation and temporal isolation leading to loss of genetic diversity.	* Bálint *et al*. (2011); * Chapman (2013); Herzog & Hadrys (2017); Johansson *et al*. (2016); Jordan *et al*. (2016); Stoks *et al*. (2014); Swaegers *et al*. (2020); Vogt *et al*. (2007)

**Fig. 3:**
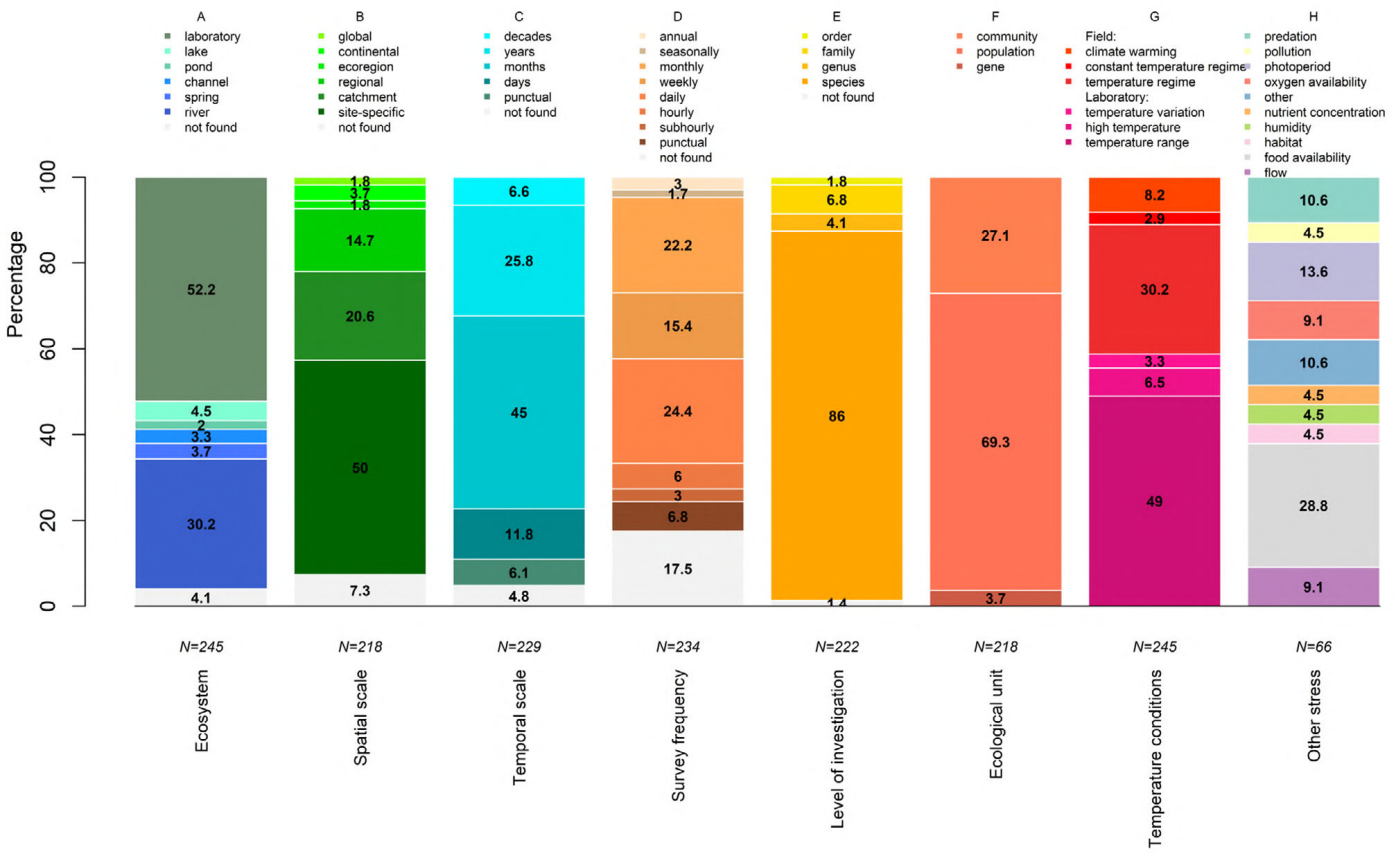
Distribution plots showing: the percentage of studies for each ecosystem type (A), spatial scale (B), temporal scale (C), survey frequency (D), level of investigation (E), ecological unit (F), temperature conditions (G) and other stresses (H).

Most studies were performed in the laboratory (*N* = 128), while among field studies lotic ecosystems were better represented (*N* = 91) compared to lentic ones (*N* = 16) (Fig. 3A). Most studies were carried out at a site‐specific (*N* = 109) or catchment scale (*N* = 45), followed by regional, ecoregional, continental and global scales (Fig. 3B). The temporal scale covered ranged mostly from months (*N* = 103) to years (*N* = 59), with fewer studies employing shorter or longer periods (Fig. 3C). Most studies used a daily or a monthly survey frequency (*N* = 57 and *N* = 52, respectively) followed by weekly or shorter intervals, although 41 studies did not provide the relevant information (Fig. 3D). Investigations at the species level were most common (*N* = 191) (Fig. 3E). Population (*N* = 151) and community (*N* = 59) studies were most prevalent, with gene‐level studies relatively rare (*N* = 8) (Fig. 3F). In laboratory studies, macroinvertebrates were commonly exposed to different temperature ranges (*N* = 120) or to temperatures that approached their upper thermal limits (*N* = 16). Field studies tended to focus on the temperature regime (*N* = 74), with a small number investigating the constant‐temperature regime of springs (*N* = 7), or climate warming (*N* = 20) (Fig. 3G). Of other stresses associated with temperature by these studies (*N* = 66), the most common were food (both quality and quantity) (*N* = 19), photoperiod (*N* = 9), presence of predators (*N* = 7), flow regime (*N* = 6) and oxygen availability (*N* = 6) (Fig. 3H).

**Fig. 4:**
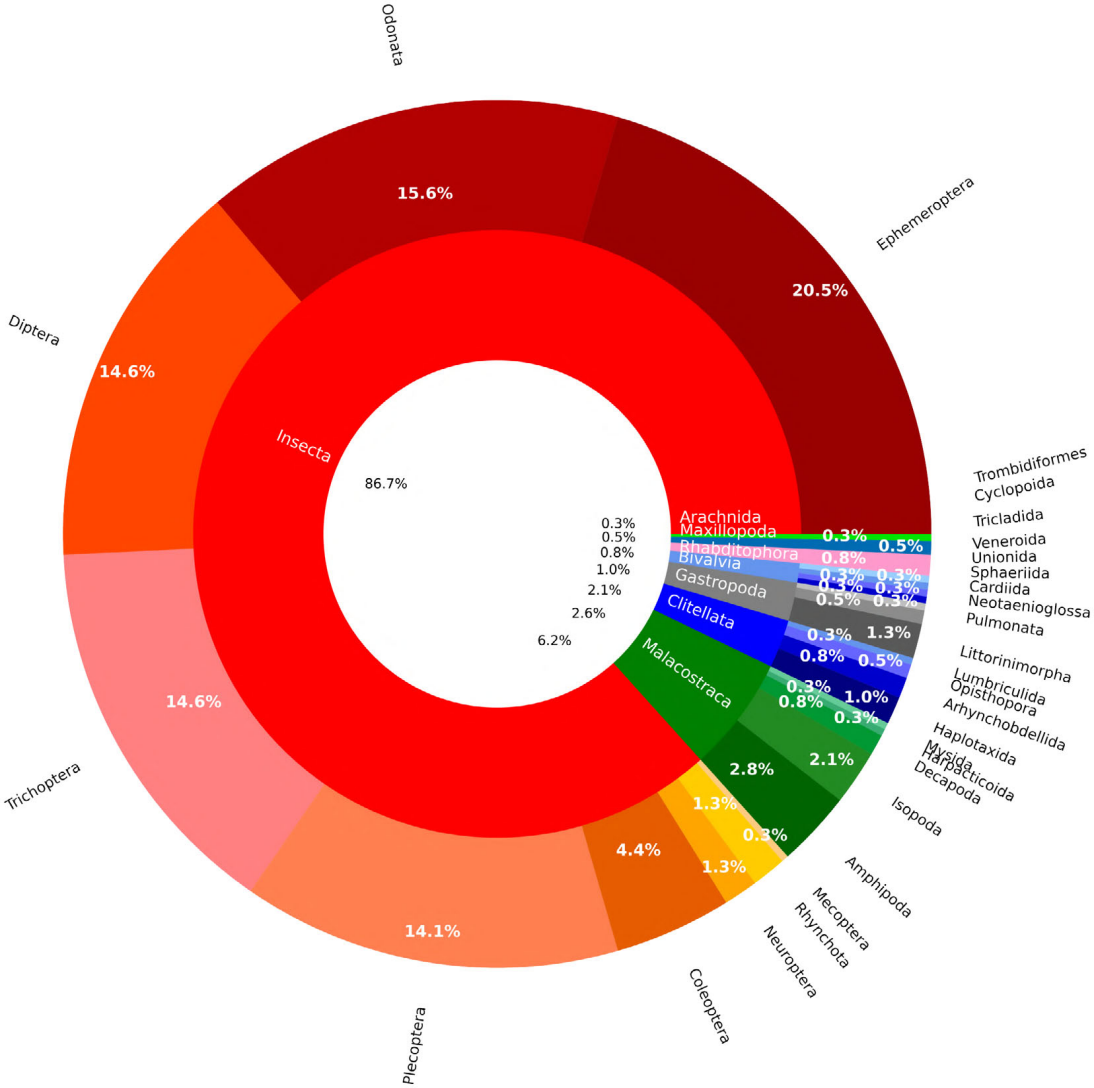
Pie‐donut chart showing the relative proportions of specific organisms investigated (*N* = 390). The internal ring refers to classes, the external ring refers to orders.

Insects were the taxon studied most often (*N* = 338) followed by Malacostraca (*N* = 24) Clitellata (*N* = 10) and Gastropoda (*N* = 8) (Fig. 4). Among insects, the majority of studies investigated Ephemeroptera (*N* = 80), Odonata (*N* = 61), Trichoptera, Diptera and Plecoptera (*N* = 57, 57, 55, respectively). Among other groups, the most popular orders were Amphipoda (*N* = 11), Isopoda (*N* = 8) and Littorinimorpha (*N* = 5) (Fig. 4).

**Fig. 5:**
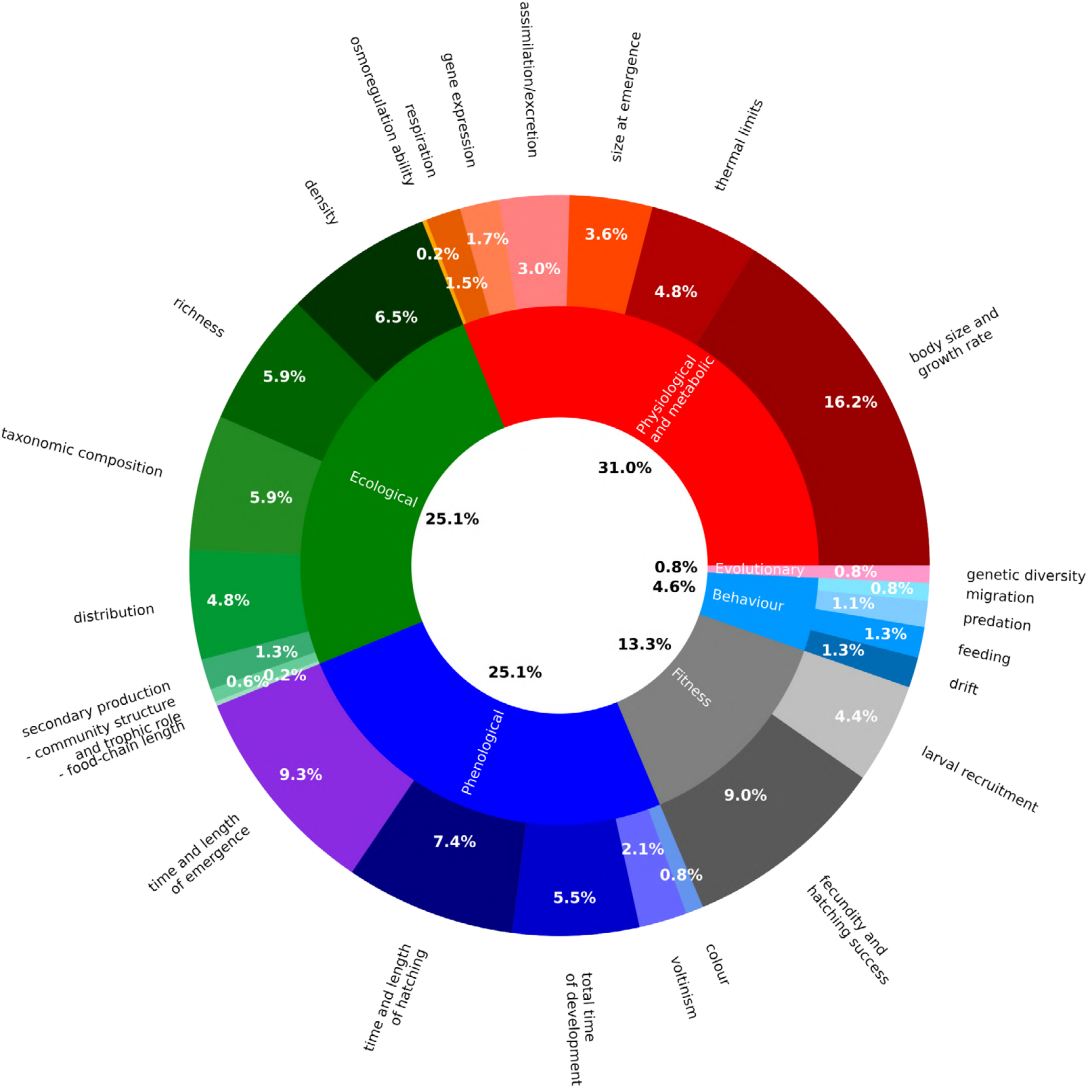
Pie‐donut chart showing the relative proportions of responses investigated (*N* = 525) grouped by type of effect. The internal ring refers to effects; the external ring refers to responses.

Among the biotic responses measures with respect to water temperature (*N* = 525, Table 3), physiological and metabolic responses were studied most extensively (*N* = 163) followed by phenological and ecological (*N* = 132), fitness (*N* = 70), and behavioural responses (*N* = 24) (Fig. 5). Among these categories, the most investigated responses were body size and growth rate (*N* = 85), time and length of emergence (*N* = 49), fecundity and hatching success (*N* = 47), time and length of hatching (*N* = 39), density, richness, and taxonomic composition (*N =* 34, 31, 31, respectively), total time of development (*N* = 29), distribution and larval recruitment (*N =* 25, 23, respectively) (Fig. 5).

**Fig. 6:**
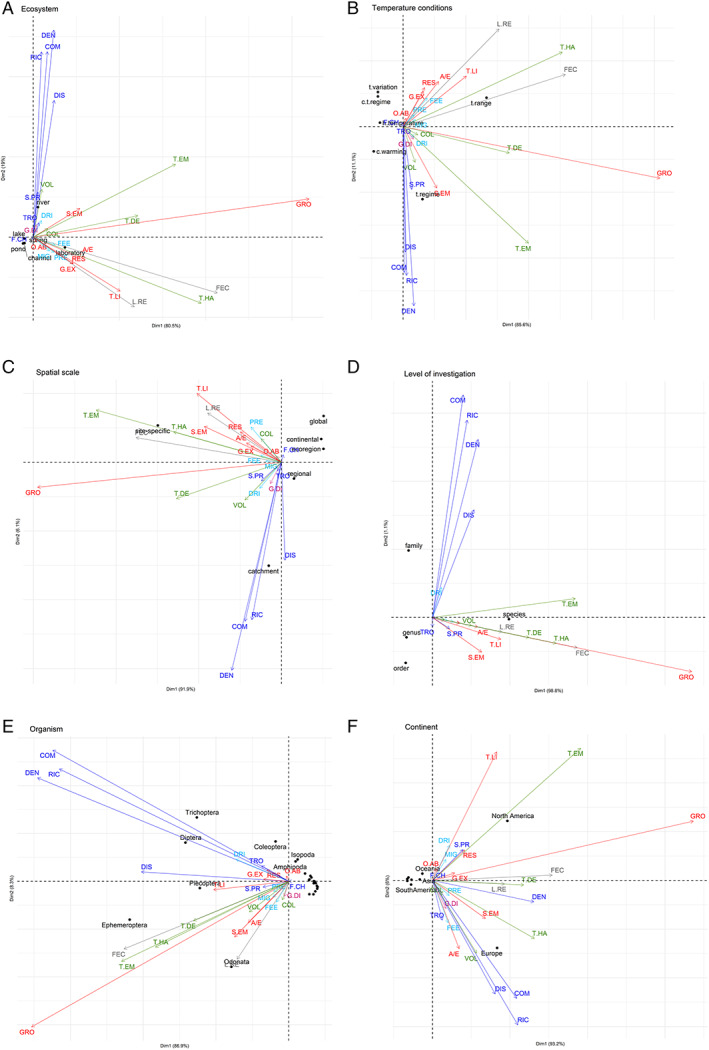
Results of principal components analysis (PCA) of recorded biotic responses of macroinvertebrates to temperature across ecosystem type (A), temperature conditions (B), spatial scale (C), level of investigation (D), organism type (E), and continent (F). Variance explained (%) is shown in parentheses. Arrows represent the frequency of responses, grouped by colour depending on the type of effects (see Table 3 and Fig. 5). Physiological and metabolic responses: gene expression (G.EX), osmoregulation ability (O.AB), respiration (RES), body size and growth rate (GRO), size at emergence (S.EM), assimilation/excretion (A/E), thermal limits (T.LI); phenological responses: total time of development (T.DE), time and length of hatching (T.HA), time and length of emergence (T.EM), voltinism (VOL), colour (COL); fitness responses: fecundity and hatching success (FEC), larval recruitment (L.RE); behavioural responses: drift (DRI), migration (MIG), predation (PRE), feeding (FEE); ecological responses: richness (RIC), taxonomic composition (COM), density (DEN), distribution (DIS), food‐chain length (F.CH), community structure and trophic role (TRO), secondary production (S.PR); and evolutionary responses: genetic diversity (G.DI).

PCA was useful to reduce the information provided by the multidimensional data set to investigate and interpret the clustering of temperature responses, examine patterns and identify potential research gaps. The results of PCA on the frequency of responses showed two main clusters, one related to biological responses (Dim1) and the other related to ecological responses (Dim2) (Fig. 6). Taxonomic composition, richness, distribution, and density were often strongly associated with each other, as were physiological and metabolic, phenological and fitness responses although there were more variable patterns of association depending on the category considered. Biological responses were generally evaluated through laboratory experiments while ecological responses were most associated with field surveys (rivers). Also, voltinism was assessed in rivers (Fig. 6A). The temperature conditions PCA exhibited the same pattern with biological responses mainly assessed exposing organisms to different temperature ranges (and to a lesser extent to temperature variation and constant temperature regime) (Dim1) while ecological responses were related to temperature regime and, secondly, climate warming (Dim2). Size at emergence, time and length of emergence, total time of development and body size and growth rate were shared between both axes (Fig. 6B). Ecological responses were studied at catchment or regional scale while biological responses (time and length of emergence, fecundity, time and length of hatching, size at emergence, larval recruitment and thermal limits) were investigated through site‐specific surveys (Fig. 6C). For biological responses, macroinvertebrates were identified at species level while most investigations were at family level for ecological studies (Fig. 6D). Among the different orders, Ephemeroptera and Plecoptera were mainly associated with studies on growth, time and length of emergence, fecundity, time and length of hatching, total time of development and thermal limits while Odonata were related to studies on larval recruitment, size at emergence, assimilation/excretion and voltinism. Trichoptera and Diptera were mainly considered in ecological studies focusing on taxonomic composition, richness, and density (Fig. 6E). Finally, ecological responses such as distribution, taxonomic composition, and richness but also responses in other categories, including voltinism, feeding and assimilation/excretion, were mainly investigated in Europe while thermal limits, time and length of emergence, secondary production, respiration and gene expression were more common in North America. Studies regarding growth, fecundity, larval recruitment, total time of development, predation and density were carried out in both continents (Fig. 6F).

### (2) Trends in thermal research during the last 50 years

**Fig. 7:**
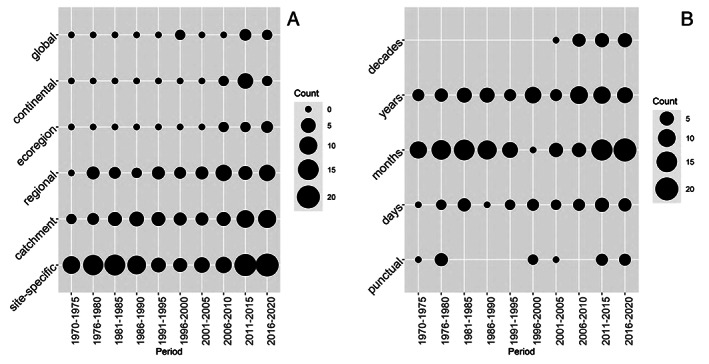
Bubble charts showing the number of studies from 1970 to 2020 that recorded responses of macroinvertebrates to temperature at specific temporal (A) (*N* = 229) and spatial (B) scales (*N* = 218).

During recent years, the spatial scale of studies has increased, especially in the last 15 years (Fig. 7A), although site‐specific studies remain most common. Similarly, long‐term investigations (decades) of the effects of temperature have appeared relatively recently (Fig. 7B), likely due to the increasing availability of long‐term biomonitoring data sets.

**Fig. 8:**
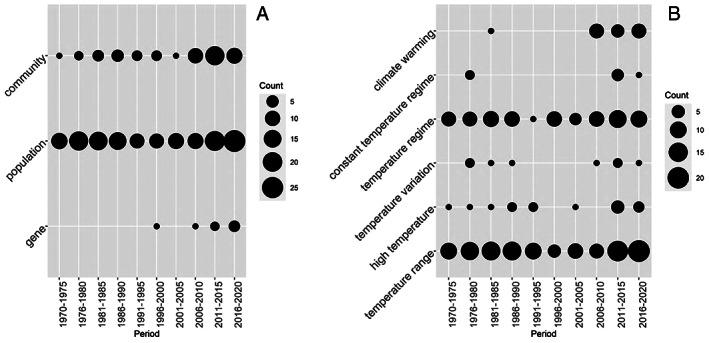
Bubble charts showing the number of studies from 1970 to 2020 that recorded responses of macroinvertebrates to temperature separated by ecological unit recorded (A) (*N* = 218) and temperature conditions (B) (*N* = 245).

Regarding the ecological unit of study, investigations of the genetic and evolutionary effects of temperature changes have appeared more recently than studies on the effects at population or community levels and remain less common (Fig. 8A). Investigations of the effects of climate change also are relatively new but represent 17% of all publications in our database since 2006 (Fig. 8B).

**Fig. 9:**
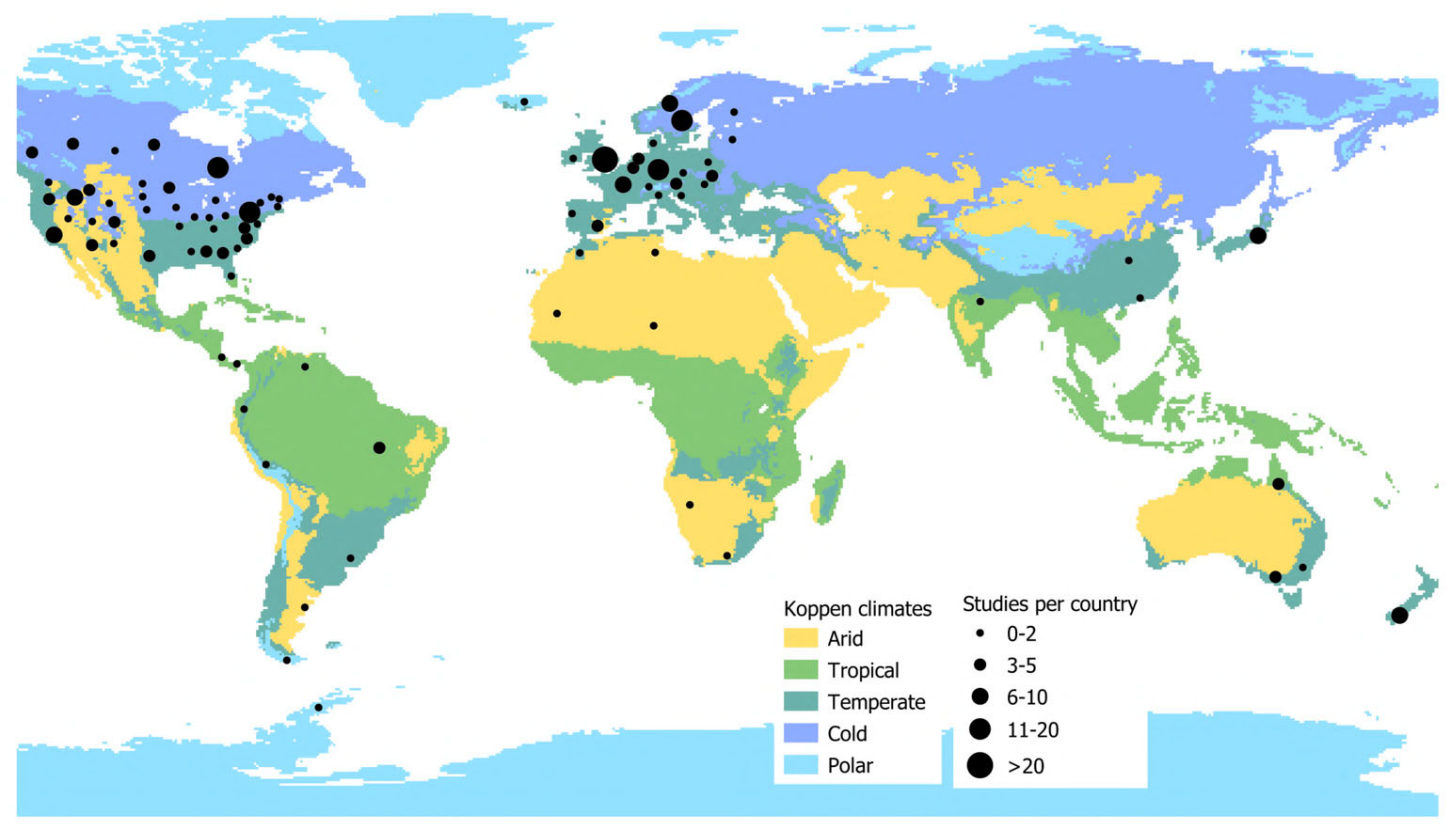
World map showing climatic regions and the number of studies per country/state (*N* = 245).

There is an unequal distribution of study sites (*N* = 245) across the different climatic regions of the world, with temperate (~57%) and cold (~28%) regions best represented, followed by tropical, arid and polar (~5%) (Fig. 9). Most studies (*N* = 192) have been carried out in Europe (~41%) and North America (~38%), with the rest of the world poorly represented (*N* = 53) (Fig. 9).

## IV. RESPONSES OF MACROINVERTEBRATES TO WATER TEMPERATURE

Table 4 provides a summary of responses of macroinvertebrates to temperature.

### (1) Physiological and metabolic effects

#### (a) Gene expression

Stressful environmental conditions such as heating induce the expression of several genes that control the activity of the heat‐shock proteins (HSPs). The reviewed papers that reported genetic responses of macroinvertebrates to temperature changes all recorded upregulation or downregulation of different genes, including HSP genes (Karouna‐Renier & Zehr, 1999; Lencioni *et al*., 2013). For example, when subjected to heat‐induced stress, *Chironomus riparius* (Diptera) did not activate or repressed some HSP genes (e.g. HSP22) while others were activated (HSP23, HSP24, HSP34, HSP27 and HSP70) suggesting that the HSP subfamily possesses remarkable functional differentiation in response to stressful temperature conditions (Martín‐Folgar *et al*., 2015). Similarly, Chou *et al*. (2018) observed that *Neocloeon triangulifer* (Ephemeroptera) larvae bred at a chronic threshold (30 °C) upregulated indicators of thermal stress (HSP90) but not genes sensitive to hypoxia [egg laying defective 9 (EGL‐9) and lactate dehydrogenase (LDH)], indicating that the upper chronic thermal limit is not set by oxygen availability. Chronic thermal stress can lead to reductions in body size and fitness through reduced food intake, which results from the upregulation of genes producing histamine and dopamine (Chou *et al*., 2018). Upregulation of HSP70 has also been observed in stenotherm species [*Lednia* sp. (Plecoptera) and *Crunoecia irrorata* (Coleoptera)] in their natural temperature range, indicating that the thermal niche they occupy may not be optimal due to other limiting factors such as biotic interactions or resource availability (Hotaling *et al*., 2020; Ebner, Ritz & von Fumetti, 2019). This challenges the assumption that the distribution of insects in cold habitats reflects evolved preferences for those temperature conditions. Teets *et al*. (2013) reported upregulation of genes involved in both glycogenolysis and gluconeogenesis in *Belgica antarctica* midges in response to heat and cold stress, suggesting that insects exposed to extreme environmental conditions mobilize carbohydrate energy stocks to allow rapid shifts in metabolism. Hotaling *et al*. (2020), studying high‐altitude stoneflies exposed to their CT_max_, identified upregulation of genes involved in the developmental transition [ATP binding cassette subfamily A member 3 (ABCA3) and hexamerins (HEXA)]. Studies on gene expression allow us to understand the physiological mechanisms underlying organismal responses to temperature changes and are imperative for correct interpretation of the causes driving biological responses at different levels, for example, to disentangle behavioural and evolutionary responses (Hotaling *et al*., 2020; Schmeller *et al*., 2018). As stated by Clarke (2003) we can identify the relationships between cellular thermal physiology and organismal physiology as well as between some macroecological patterns and temperatures, however, we are still unable to relate thermal physiology to ecology at the community scale, despite this link likely being a strong determinant of life‐history traits, food‐web dynamics, and biological diversity.

#### (b) Osmoregulation ability

Temperature affects the regulation of haemolymph osmotic and ionic concentrations in invertebrates. In general, increasing temperatures increase ion transport rates (Orr & Buchwalter, 2020). We found only one study on macroinvertebrate osmoregulation in which Colburn (1983) observed that larvae of *Limnephilus assimilis* (Trichoptera) exposed to a wide salinity range (0–25%) could complete their development at low temperature because cellular Cl^−^ and Na^+^ were maintained at low concentrations. On the contrary, at high temperatures (for example in hydrothermal water) they were unable to control Cl^−^ intake, leading to lower survival and decreased ability to complete development.

#### (c) Respiration

As for all biological processes, respiration rate is positively correlated with temperature (Sinsabaugh, 1997), hence higher temperatures enhance the oxygen consumption of invertebrates, as shown by Bergström *et al*. (2010) for species in lake sediments. At higher temperatures, larger amounts of energy are required for metabolic maintenance, for both respiration and assimilation, compared to at the thermal optimum (Sweeney & Vannote, 1978; Vannote & Sweeney, 1980). However, due to the decrease in oxygen solubility with increasing temperature, oxygen availability is reduced simultaneously with this greater respiratory requirement (Forster, Hirst & Atkinson, 2012). The sensitivity of species to this decrease in oxygen availability varies depending on the taxon. Some species such as *Leuctra hippopus* (Plecoptera) and *Asellus aquaticus* (Isopoda) can maintain a constant respiration rate independent of ambient oxygen levels (below a critical limit) (Rotvit & Jacobsen, 2013; Kim *et al*., 2017), whereas others, such as *Isoperla* spp. (Plecoptera) show a higher oxygen consumption with increasing temperature and a respiratory rate that is proportionally greater in larger species (Modlin & Jayne, 1981). Some chironomid species [e.g. *Chironomus anthracinus* (Diptera)] can shift from aerobic metabolism to partially anaerobic (Hamburger, Dall & Lindegaard, 1994) as temperature increases (from 2 to 20 °C).

#### (d) Body size, growth rates and size at emergence

Growth and adult body size depend on several processes regulated by temperature such as rates of ingestion, assimilation, metabolism, and excretion. Sweeney & Vannote (1978) conceptualized this temperature–growth–size relationship in their TEH according to which maximum adult size is achieved at a thermal optimum while outside this optimal range body size is reduced. Several studies have demonstrated that higher temperatures can cause acceleration of metabolism and consequently lower investment in growth, leading to premature adult development. On the contrary, at low temperatures, metabolic activity is slowed down, allowing a greater proportion of adult tissue maturation (Vannote & Sweeney, 1980; Sweeney & Vannote, 1978, Brittain, 1983; Rempel & Carter, 1987; Sweeney, Vannote & Dodds, 1986*a*). The size–temperature relationship is generally assessed in laboratory studies, in which organisms are bred at a constant temperature and measured and weighed at frequent intervals (typically 1–3 days). Morphological traits considered include total length, head capsule width, thorax length, pronotal length, wing length, leg length, antennal length, and body mass, depending on the taxon and developmental stage. The observed temperature–body size relationship often follows an exponential curve (Brittain, 1983; Giberson & Rosenberg, 1992*a*; Sweeney & Vannote, 1984; Reynolds & Benke, 2005; Rempel & Carter, 1987). Growth rates are calculated from the change in body size (both length and mass) for specific intervals of the developmental period. The temperature–body size relationship can also be studied in relation to sex and or life‐cycle phase. Some experiments have shown that at high temperatures females reach smaller adult sizes than males suggesting that somatic growth is traded off against reproductive capacity (Rempel & Carter, 1987; McKie, Cranston & Pearson, 2004) while in other studies sexual dimorphism appears unaffected by temperature, with other factors such as sexual selection or fertility playing an important role (Lande, 1980; Encalada *et al*., 2019).

Although some evidence shows that higher temperatures lead to smaller adult body size (in agreement with the TEH), other studies on both terrestrial and aquatic ectotherms found a maximal adult size only at the coldest extreme of the species' thermal tolerance range and not at some intermediate temperature (conflicting with the TEH) (Atkinson, 1994, 1995; Sweeney *et al*., 2018). Such observations led to the development of the temperature size rule (TSR) (Atkinson, 1994), which was reformulated by Kingsolver & Huey (2008) as ‘hotter is smaller’. It seems to represent a special case of Bergmann's (1847) rule according to which populations/species of larger size are found in colder environments. Subsequent studies (Forster *et al*., 2012; Horne, Hirst & Atkinson, 2015), reviewing a large number of temperature–body size experiments involving freshwater, marine and terrestrial species have confirmed the TSR hypothesis and showed that warming‐induced reductions in adult body size are larger for aquatic ectotherms than for terrestrial ones. Recently studies have begun to investigate the drivers that explain the TSR rule. Although temperature responses appear to be outcomes of phenotypic plasticity, latitudinal size gradients could depend also on genetic factors (Horne *et al*., 2015). Insect temperature–body size trends observed across latitudinal clines have not been replicated across altitudinal gradients (Horne, Hirst & Atkinson, 2018). TSR explanations have focused on physiological processes (such as oxygen limitation and resource availability) and responses (shorter developmental times due to higher mortality at higher temperatures), and on ecological and evolutionary mechanisms (adaptation to temperature to maximize fitness). Many of these studies support oxygen as a significant factor (Forster *et al*., 2012; Verberk *et al*., 2021). The higher cost of oxygen uptake in warmer water and the greater demands on large bodies to maintain aerobic scope in warmer environments both play important roles in determining adult size (Woods, 1999) and could explain the different temperature–size responses between aquatic and terrestrial organisms (Forster *et al*., 2012) and across latitude and altitude (Horne *et al*., 2018).

#### (e) Assimilation/excretion

High temperatures cause an increase in the fraction of energy needed for metabolism maintenance (Sweeney & Vannote, 1978), which requires greater food consumption and leads to faster gut clearance (Zimmerman & Wissing, 1978). At high temperatures, some organisms, such as *Hydropsiche betteni* (Trichoptera), seek better‐quality food (animal material or algae instead of detritus) to cope with higher energy demands (Fuller & Fry, 1991), whereas *Chironomus riparius* (Diptera) and *Mesogomphus lineatus* (Odonata) do not show dietary changes depending on temperature (Péry & Garric, 2006; Pandian, Mathavan & Jeyagopl, 1979). Food uptake and assimilation rates increase with temperature up to the thermal optimum (Culler, McPeek & Ayres, 2014; Van Doorslaer & Stoks, 2005*a*; McCauley, Hammond & Mabry, 2018; Stoks, Swillen & De Block, 2012; Pandian *et al*., 1979; Péry & Garric, 2006).

#### (f) Thermal limits

Thermal limits are usually measured in laboratory studies (Fig. 6A) by exposing organisms to temperatures increasingly distant from their optimal temperature range (Sherberger *et al*., 1977). Organismal death occurs when the water temperature reaches the critical thermal limits (Sherberger *et al*., 1977; Rogowski & Stewart, 2016; Chou *et al*., 2018; Sweeney *et al*., 1986*a*; Rosillon, 1988). The upper thermal tolerance can be determined by the LT_50_ test: this threshold represents the lethal upper temperature at which 50% of individuals die in a specified time. By contrast, the incipient lethal temperature (ILT) thermal limits are based on the most extreme temperatures at which 50% of the test organisms survive indefinitely after being transferred from an acclimation temperature directly into a constant‐temperature tank where time to death is measured (Brett, 1952). A less time‐consuming approach that requires smaller samples is the critical thermal method (CTM) which consists of assessing the behavioural stress response, defined as the ‘arithmetic mean of collected thermal points at which locomotor activity becomes disorganized to the point at which the organism loses its ability to escape conditions that will promptly lead to its death’ (Lowe & Vance, 1955, p. 2). For aquatic macroinvertebrates, the response includes the inability to remain attached to the substrate and hyposensitivity to stimuli. All these methods have been employed in studies of thermal biology and a review focused on terrestrial animals comparing these different approaches is available (Lutterschmidt & Hutchison, 1997). For aquatic insects, the upper thermal limit evaluated at 96 h (96‐LT_50_) and the CT_max_ are related by a significant positive linear relationship, establishing the CTM method for use (Dallas & Ketley, 2011). There have been various attempts to define the thermal threshold of different aquatic macroinvertebrate taxa based on laboratory experiments on individual species or using the relationship between the macroinvertebrate assemblage and the temperature regime of the water bodies where they are found. Stewart *et al*. (2013) defined the upper thermal tolerance of 13 taxonomic groups (mainly at order level) of southwestern Australian macroinvertebrates by reviewing the existing literature and measuring LT_50_ for four key species. Dallas & Rivers‐Moore (2012), using the CTM, determined the upper thermal limits for 27 families of South African macroinvertebrates. Polato *et al*. (2018) and Shah *et al*. (2017) quantified CT_max_ and CT_min_ of 62 EPT species from Colorado (USA) and the Andes, showing that the tropical (Andean) species had a narrower thermal tolerance than the temperate ones. Niedrist & Füreder (2020) redefined the temperature optima and thermal ranges for different species of EPT and chironomids (Diptera) using regression models for long series of water temperature data and showed that alpine benthic communities had moved to higher altitudes in the last decade due to glacial retreat.

### (2) Phenological effects

Phenological responses are related to the life cycle and the duration of developmental stages (Vannote & Sweeney, 1980; Ward & Stanford, 1982). Temperature influences the total development period as well as the number of annual cohorts, and the timing of hatching and emergence (Woods, Kaz & Giam, 2021). Understanding how temperature regulates the life history of a taxon could allow us to predict its phenological responses to climate change (Dingemanse & Kalkman, 2008; McCauley *et al*., 2018). The available life‐history studies involve both laboratory and field experiments (Fig. 6A), with samples of macroinvertebrates observed regularly to assess the overall duration of development or that of specific stages. Moreover, the organisms are counted and/or measured (length and biomass) to understand the influence of temperature on each instar and the number of generations produced per year. In insects, phenological responses of aquatic stages are monitored by assessing embryonic time (from egg deposition to hatching), larval time (from hatching to emergence) or the entire aquatic period (from egg deposition to emergence) (Brittain, 1977; Humpesch, 1980*a*; Giberson & Rosenberg, 1992*b*). The developmental period of macroinvertebrates can vary from a few months up to 3 years. A species may be semivoltine, univoltine, bivoltine, trivoltine or polyvoltine where the number of broods in 1 year is <1, 1, 2, 3 or >3, respectively (Hynes, 1970). Some species can modify their developmental period in response to temperature (voltinism plasticity) (Braune *et al*., 2008).

#### (a) Total time of development

Several studies have shown that increasing temperature leads to shorter developmental time. Sweeney *et al*. (1986*a*) showed that the larval development of *Leptophlebia intermedia* (Ephemeroptera) is shorter at higher temperatures and Sweeney & Vannote (1984) reported the same for *Cloeon triangulifer* (Ephemeroptera). Other studies confirmed that developmental time, within the tolerance range, decreases with increasing temperature for eurythermal species (Sarvala, 1979; Frouz, Ali & Lobinske, 2002; Bayoh & Lindsay, 2003; Imholt *et al*., 2010; McCauley *et al*., 2015). By contrast, for stenothermal species like *Soyedina carolinensis* (Plecoptera), the shortest developmental time (~92 days) was observed at an optimal temperature (10 °C), increasing at both higher (15 °C) and lower temperatures (5 °C) (~109 and 141 days, respectively) (Sweeney, Vannote & Dodds, 1986*b*). The same pattern was observed for *Eukiefferiella ikleyensis* (Diptera), with the shortest larval stage at 14 °C compared to both higher (18 °C) and lower (9 °C) temperatures (~71, 74 and 110 days, respectively) (Storey, 1987). The relationship between temperature and developmental time for stenothermal species can be described by a parabolic curve (Sweeney *et al*., 1986*b*; Elliott, 1987) while for eurythermal species the trend typically follows a negative exponential model (Marten, 1990) or an inverse asymptotic correlation (McKie *et al*., 2004). Frouz *et al*. (2002) reported that under increasing temperatures chironomid males developed faster than females.

#### (b) Time and length of hatching

Temperature is a crucial determinant of invertebrate hatching time. In general, temperatures far from the optimal range induce diapause (Danks, 1987), an adaptation evolved by some organisms to extend the embryogenesis period until the environmental conditions are suitable (Pritchard, Harder & Mutch, 1996). The relationship between hatching time and temperature follows a decreasing trend best described by a power function (Brittain, 1977, 1982; Bohle, 1972; Brittain & Campbell, 1991; Elliott, 1986, 1972, Giberson & Rosenberg, 1992*b*; Humpesch, 1980*a*,*b*; Mendonça *et al*., 2018), or a hyperbolic power function (Elliott, 1978; Friesen, Flannagan & Lawrence, 1979), at least within the temperature tolerance range. Hatching time decreases at higher temperatures, more sharply in warm‐adapted species such as Odonata than in cold‐adapted species such as Plecoptera (Pritchard *et al*., 1996; Bouton, Iserbyt & Van Gossum, 2011). Diapause is longer at high temperatures for stenothermal species; eurytherms can survive low temperatures by remaining dormant (Pritchard *et al*., 1996). Embryonic period is positively correlated with egg size and the relationship between these does not seem to change with temperature (in the range 10–25 °C) in both univoltine and multivoltine species of mayflies, stoneflies, caddisflies, Coleoptera, Hemiptera and dragonflies (Gillooly & Dodson, 2000*b*).

#### (c) Time and length of emergence

Increasing temperatures typically lead to earlier emergence of insects (Nebeker, 1971; Rempel & Carter, 1987; Vannote & Sweeney, 1980; McCauley *et al*., 2018). In aquatic environments characterized by a variable temperature regime, the pivotal factor regulating emergence is temperature while in constant‐temperature habitats photoperiod plays a major role (Ivković *et al*., 2013). Water temperature is the primary driver that determines the timing of emergence for holometabolous insects (where the pupae are submerged) while other variables (such as humidity and air temperature) are involved for hemimetabolous insects (Trottier, 1973*b*; Ivković *et al*., 2013). In recent decades the emergence of Odonata adults takes place earlier in the year due to increased temperatures. According to Hassall & Thompson (2008), British Odonata have advanced their emergence by about 1.15 days per decade and 3 days per degree between 1960 and 2004, showing a phenological response to climate change similar to those observed for terrestrial taxa (Lepidoptera, amphibians, birds and plants) (Sparks, Jeffree & Jeffree, 2000). A similar pattern was reported for Dutch Odonata (Dingemanse & Kalkman, 2008) and the German population of *Gomphus vulgatissimus* (Odonata) (Richter *et al*., 2008). Although Odonata is the best‐studied group in terms of temperature‐related emergence, there are similar findings for EPT and Diptera (Nebeker, 1971; Čmrlec *et al*., 2013; Dickson & Walker, 2015; Chacón, Segnini & Briceño, 2016; Cheney *et al*., 2019; Nyquist, Vondracek & Ferrington, 2020).

#### (d) Voltinism

In response to different temperature conditions, aquatic macroinvertebrates can show phenotypic plasticity that can speed up or slow down the development of adaptive strategies (Pritchard *et al*., 1996). For example, some stoneflies (e.g. *Nemoura cinerea*) are able to shift from a univoltine to a semivoltine life cycle when the eggs are exposed to a low temperature (10 °C) (Brittain, 1974). Under increasing temperature, some stoneflies (e.g. *Leuctra nigra*) and mayflies (e.g. *Ephemerella danica*) shift from semivoltine to univoltine, showing highly plastic phenology (Elliott, 1987; Everall *et al*., 2015). Many species have a synchronous life cycle coordinated by water temperature (Humpesch, 1980*a*; Sweeney & Vannote, 1984). For example, *Beatis alpinus* (Ephemeroptera) has a trivoltine/bivoltine or univoltine life cycle depending on altitude (Humpesh, 1979; Erba, Melissano & Buffagni, 2003) although Bottová, Derka & Svitok (2013*b*) found asynchronous life cycles in specimens maintained at constant temperature conditions. By contrast, a recent study carried out in a karstic spring of the Western Carpathians (Beracko & Revajová, 2019) investigating more than 40 benthic species did not support the proposal that constant water temperature promotes asynchronous life cycles and reported different phenological responses. Some Plecoptera species (e.g. *Protonemura auberti* and *Leuctra albida*) had an additional winter cohort instead of entering diapause, other species from various orders [e.g. *Gammarus fossarum*, (Amphipodae) *Rhyacophila tristis* (Trichoptera) and *Protonemura austriaca* (Plecoptera)] showed an unchanged or even a longer nymphal development while others maintained fixed voltinism [*Ephemerella mucronate* (Ephemeroptera), *Isoperla sudetica* (Plecoptera)]. Odonata species tend to show a clear voltinism gradient along latitude and temperature clines: voltinism decreases from Southern to Northern Europe ranging from one generation every 1–2 years in the south to one generation every 5 years in the north (Söndgerath, Rummland & Suhling, 2012), indicating that higher temperatures correlated with increasing voltinism (Braune *et al*., 2008). Univoltine species are likely to be negatively impacted by increases in temperature extremes whereas multivoltine species are likely to be advantaged (Rivers‐Moore, Dallas & Ross‐Gillespie, 2012).

#### (e) Colour

McCafferty & Pereira (1984) noted that in larvae of *Hexagenia limbata* and *Stenacron interpunctatum* (Ephemeroptera) the colour of the body and wings, as well as the spotting pattern, depended on the temperature regime of the water in which larvae developed. The colour of the compound eyes and legs was independent of temperature. Abbott (2013) conducted experiments on female *Ischnura elegans* (Odonata), a three‐colour polymorphic species, to investigate whether colour polymorphism was correlated with thermal performance. He found that life‐history traits varied across colour morphs, suggesting that thermal performance was more associated with morphospecies rather than local thermal adaptation.

### (3) Fitness effects

#### (a) Fecundity and hatching success

In most invertebrates, fecundity is directly proportional to female body size (Rempel & Carter, 1987). High temperatures reduce the capacity of organisms to exploit resources from the ecosystem (Marten, 1990), leading to a decrease in the energy available for egg production, and thus to lower fecundity (Sweeney & Vannote, 1978; Rempel & Carter, 1987; Rosillon, 1988; Pritchard *et al*., 1996; Dallas & Ross‐Gillespie, 2015). Increasing temperature also leads to faster hatching and lower egg survival (Bouton *et al*., 2011), partly due to a greater risk of infection by fungi and bacteria (Harvell *et al*., 2002; Marcogliese, 2016). In response to stressful temperature conditions, aquatic insects face a trade‐off between growth and reproduction. According to the TEH, fecundity varies with altitude and latitude, declining as temperatures move away from the optimum. For example, Van Doorslaer & Stoks (2005*b*), studying two congeneric damselflies *Coenagrion hastulatum* and *C. puella* (Odonata) widespread in northern and central Europe respectively, identified the evolution of latitudinal compensation to low temperature in line with predictions of the TEH, but only at the embryogenic stage and not at the larval stage. This observation stresses the importance of assessing thermal responses at different life‐history stages. Each species has a specific thermal threshold for egg hatching and development, which will affect both population size and species distribution (Elliott, 1988; Lambret, Hilaire & Stoks, 2017). Optimal temperatures promote the largest broods and eggs, higher hatching success and greater reproductive success (Bovill, Downes & Lancaster, 2015), while higher temperatures have significantly negative effects on egg survival and overall fitness (Starr & McIntyre, 2020). Low temperatures prolong dormancy and delay hatching (Danks, 2002; Lencioni, 2004).

#### (b) Larval recruitment

With increasing temperature the nymph recruitment increases while growth rates increase exponentially (Wright, Mattice & Beauchamp, 1982; Humpesch, 1980*a*,*b*; Strange, 1985; Marten, 1990; Corkum & Hanes, 1992; McCauley *et al*., 2018; Chavez *et al*., 2015; Lencioni *et al*., 2013; Ingram, 1976; Van Doorslaer & Stoks, 2005*b*). Some studies show that survival rates differ between the sexes, suggesting an interaction between sex and temperature. Other factors may also play important roles in larval recruitment, such as the ability to reproduce parthenogenetically (Wright *et al*., 1982).

### (4) Behavioural effects

#### (a) Migration and drift

Temperature varies seasonally and within a waterbody, especially in rivers and deep lakes. As ectotherms, aquatic macroinvertebrates must maintain their metabolic and physiological processes at levels high enough to survive and reproduce (Vannote & Sweeney, 1980). Aquatic species can track suitable thermal niches by dispersal through drift or active swimming, with drift being the most common dispersal type in rivers (Waters, 1965). There are two types of drift: catastrophic (mainly due to disruptive floods or hydropeaking as well as drought, high temperature and pollution) and behavioural, occurring when macroinvertebrates voluntarily leave their substrate in response to stress conditions that include temperature, predation or resource scarcity (Muller, 1954; Waters, 1965; Wiley & Kohler, 1980). A variety of studies have recorded distinct drift in benthic invertebrates exposed to thermal and discharge waves caused by sudden water release from hydropower plants, with catastrophic drift due to hydropeaking and behavioural drift caused by thermopeaking. Chironomidae, Simuliidae (Diptera) and Baetidae (Ephemeroptera) resulted the most abundant drifting taxa (Bruno *et al*., 2012; Carolli *et al*., 2012; Schülting, Feld & Graf, 2016). Temperature can influence drift, for example, Wojtalik & Waters (1970) observed that increased temperature resulted in nocturnal drift in *Baetis vagans* (Ephemeroptera) but not *Gammarus pseudolimnaeus* (Amphipoda), while at constant temperature conditions neither species drifted. Scherr, Wooster & Rao (2010) reported greater drift in the mayfly *E. alberta* at a high temperature (28 °C). High water temperatures can also promote emergence events as shown by Trottier (1973*b*) for the climbing speed of *Anax junius* (Odonata).

#### (b) Predation

High temperatures may disproportionately influence organisms at higher trophic levels which are more strongly affected by alterations of energy fluxes across the food web (Vasseur & McCann, 2005; Gilman *et al*., 2010). Thus, theoretically predators may be more vulnerable to increasing temperatures than their prey. However, McKie & Pearson (2006) showed that predation of Australian chironomids by *Australopelopia prionoptera* (Diptera) was not influenced by different temperatures (12, 18 and 26 °C). This suggests that in macroinvertebrates characterized by broad physiological tolerances the predator–prey relationship may be unaffected by temperature. Thermal shocks did not alter predation of Ephemeroptera (Sherberger *et al*., 1977), with mortality of individuals of *Isonychia* sp. at 33 °C for 30 min due to the presence of a predatory fish (*Cottus carolinae*) similar to that for the control group (14 °C). By contrast, Smolinský & Gvoždík (2014) found that during daily temperature extremes predation rates on newt larvae diminished, despite increased predator (dragonfly larvae) movement. Predation pressure may be lower at high seasonal temperatures or where fauna have a broad thermal tolerance range (Hildrew & Giller, 1994; Reice, 1994; McKie & Pearson, 2006).

In boreal freshwater systems, predator–prey interactions are particularly sensitive to thermal changes due to the simpler trophic web and to the presence of stenothermal species (Thompson, 1978; Moore & Townsend, 1998). For example, Kishi *et al*. (2005), studying the trophic chain of a boreal stream composed by a predatory fish (*Salvelinus malma*), an herbivorous caddisfly (*Glossosoma*) and periphyton, observed that thermal habitat alteration can change food‐web structure *via* combinations of direct and indirect trophic interactions. Indeed, at high temperature (21 °C) *Glossosoma* larvae were promoted due to both the lower salmonid predation and the greater availability of periphyton. On the other hand, high temperature can reduce the ability to build cases in Trichoptera larvae due to the high energetic cost (Mondy *et al*., 2011) leaving them more exposed to predators. For example, Rogowski & Stewart (2016) observed decreased retreat building and higher mortality in *Leptonema* sp. (Hydropsichae, Trichoptera) at 22 °C.

#### (c) Feeding

A variety of studies have shown that key consumers in freshwater ecosystems change their feeding behaviour depending on temperature conditions. Metabolism is enhanced by increased temperatures, and this generates the requirement for a greater energy intake (Vannote & Sweeney, 1980). Greater feeding efficiency can be achieved either by targeting resources that are more easily assimilated or by seeking higher quality food. For example, in a geothermal stream network characterized by a large temperature range (5–23 °C), at warmer temperatures (20 °C) the snail *Radix balthica* (Gastropoda) shifts to a more specialized diet while the black fly *Simulium aureoum* (Diptera) switches from active collection of sessile diatoms to passive filter‐feeding on motile diatoms. On the contrary, the chironomid *Eukiefferiella minor* (Diptera) becomes more generalist at warmer temperatures (Gordon *et al*., 2018). Diet and temperature may interact: food quality influences both growth rates and body size in shredders, scrapers and grazers of EPT and Diptera (Fuller & Fry, 1991; Giberson & Rosenberg, 1992*a*; Rosillon, 1988; Storey, 1987; Sweeney *et al*., 1986*a*,*b*). The interactions among food, temperature, developmental time and fecundity suggest that the TEH should be adapted to include both food quality (Rosillon, 1988) and availability.

### (5) Ecological effects

Ecological responses to temperature involve the whole macroinvertebrate community and include relationships among the taxa and their trophic roles, as well as the structure of the community itself.

#### (a) Community richness, taxonomic composition, and density

Macroinvertebrate community composition varies with temperature at both micro‐ and macro‐geographic spatial scales; temperature affects the selection and maintenance of different species in water bodies (Vannote & Sweeney, 1980). A clear trend of increasing richness occurs with increasing temperature (along both altitudinal and latitudinal gradients); Castella *et al*. (2001) showed this pattern for glacier‐fed streams across Europe. In the Po catchment (Italy), a clear altitudinal pattern in macroinvertebrate community composition was identified. Assemblages inhabiting high‐altitude sites were characterized mostly by Plecoptera, Trichoptera, Coleoptera, and Diptera, whereas macroinvertebrate communities inhabiting lowland sites included mostly non‐insect orders such as Clitellata, Gastropoda and Bivalvia. At the temporal scale, annual thermal variability promotes seasonal dissimilarity in macroinvertebrate assemblages (Vannote & Sweeney, 1980; Ward & Stanford, 1982; Arai *et al*., 2015) while inter‐annual water temperature variations affect community composition. For example, Fornaroli *et al*. (2020) found that in Northern Italy inter‐annual flow and temperature regime variations affected community richness with higher alpha diversity in warmer years but lower EPT taxa abundance. Extreme temperatures can cause decreases in both species numbers and density (Vannote & Sweeney, 1980; Voelz, Poff & Ward, 1994; Glazier, 2012; Arai *et al*., 2015) inducing shifts in community composition (Arim, Bozinovic & Marquet, 2007*a*).

Climate warming may promote eurythermal and generalist species with a consequent expansion of these less‐specialized macroinvertebrate communities (Domish, Jahnig & Haase, 2011). This is likely to affect springs and small streams more than large rivers (Haidekker & Hering, 2008). Increasing temperatures led to the upstream spread of eurythermal and rheophilic species from subalpine levels, causing homogenization of macroinvertebrate communities, especially for EPT (Timoner *et al*., 2020). Increasing temperatures thus result in movement of species upstream and an increase in invasive species (Jourdan *et al*., 2018).

#### (b) Distribution

Temperature affects species distributions both positively and negatively: some can be expected to increase their distribution in response to climate warming (‘winning’ species as defined by Domish *et al*., 2011) while others become more restricted (‘loser’ species) (Sweeney & Vannote, 1978; Arai *et al*., 2015; Jacobsen *et al*., 2014; Besacier *et al*., 2019). Adaptation to a specific temperature range restricts species zonation to particular ecological niches along the temperature gradient (Sweeney & Vannote, 1978; Arai *et al*., 2015).

In recent years, attempts have been made to predict the distribution of freshwater communities at national, continental, and global spatial scales by applying predicted air temperatures (sometimes together with precipitation predictions) to models using long‐term aquatic monitoring data sets. Several indicators have been developed to assess the sensitivity and vulnerability of aquatic communities to climate change (Hering *et al*., 2009; Conti *et al*., 2014; Sandin *et al*., 2014; Mustonen *et al*., 2018). Among the environmental drivers that regulate taxa distribution, climatic drivers contribute substantially at a broad geographic scale but are insufficient to explain local community dynamics at catchment scale, where other variables such as habitat, geomorphology and land‐use features play an important filtering role (Poff *et al*., 2010; Feld & Hering, 2007). Other factors influencing the macroinvertebrate community at the local scale include species thermal limits, adaptation capacity, drift propensity, resource use and interspecific interactions (Glazier, 2012).

#### (c) Food‐chain length

The amount of energy available in an ecosystem influences its food‐web structure (Odum, 1968) and sets an upper limit to the length of the food chain (Lindeman, 1942; Hutchinson & MacArthur, 1959). Two theories regarding the relationship between temperature and food‐chain length have been proposed. According to the metabolic theory (Arim, Marquet & Jaksic, 2007*b*), body size and food‐chain length are inversely correlated with environmental temperature: at high temperatures, metabolic demand increases so that lower levels of the trophic web consume more energy and energy flow to upper trophic levels is reduced. According to the thermal tolerance hypothesis (Brock, 1985), biochemical similarity among the organisms that constitute a specific ecological community will lead to similarity in their thermal tolerance. Therefore, below the upper thermal limit, the food‐chain length is independent of temperature while close to the limit it is considerably reduced (Brock, 1985). Glazier (2012) showed that in springs (characterized by constant temperature and flow regimes), food‐chain length decreases with increasing temperature, but the decline is not linear, broadly in support of the thermal tolerance hypothesis.

#### (d) Community structure and trophic role

Temperature changes in freshwater ecosystems potentially alter macroinvertebrate community structure, modifying trophic interactions within the aquatic food web. Taxon‐specific trait information (www.freshwaterecology.info database) can be used to investigate the mechanisms through which temperature affects community structure. For example, Jourdan *et al*. (2018) used long‐term data (10–30 years) on macroinvertebrates from several streams in the UK, Germany, Finland, and Latvia to show that the composition of functional feeding groups was strongly impacted by warming temperatures and more intense precipitation events. In particular, grazers and scrapers appeared especially vulnerable at higher temperatures, as predicted by Pyne & Poff (2017) for the macroinvertebrate communities of the western USA. Trait information related to feeding, substrate and habitat specializations proved critical to understanding the responses of macroinvertebrates to temperature changes in Sweden (Sandin *et al*., 2014).

#### (e) Secondary production

The metabolic theory asserts that secondary production will be relatively temperature invariant, recognizing resource supply as the sole driver, and this has been validated by studies carried out in Iceland's geothermal streams (Nelson *et al*., 2017; Junker *et al*., 2020). However, basal resource dynamics depend on many variables including light, nutrient availability and temperature so apparent relationships between temperature and production can be explained by the positive effects of temperature on resource supply (Junker *et al*., 2020). Inland waters are heterotrophic ecosystems in which secondary production is strongly supported by allochthonous organic matter rather than by internal primary production. Climate change‐related mechanisms may increase the inputs of allochthonous dissolved organic carbon (Pagano, Bida & Kenny, 2014; Porcal *et al*., 2009). In addition, primary production is strongly dependent on water temperature regime (Demars *et al*., 2011; Padfield *et al*., 2017) making a general trend of increased resource availability likely with global warming. However, in addition to an increased supply of both autotrophic and heterotrophic food resources at higher temperatures, decay rates of organic matter will also be accelerated, thus decreasing its availability to consumers (Rempel & Carter, 1986). In aquatic environments characterized by constant temperatures (springs), stonefly secondary production was found to be very high, possibly due to reliable resource supply due to stable temperature (Bottová *et al*., 2013*a*) and flow regimes (Zimmerman & Wissing, 1978).

### (6) Evolutionary effects

#### (a) Genetic diversity

Temperature can also have effects at an evolutionary scale, causing thermal divergence in populations of the same species, promoting genetic diversity, and leading to temporal segregation. For example, temperate and tropical populations of the chironomids *Echinoclaudius martini* and *Australopelopia pronioptera* (Diptera) have diverged in developmental time; moreover, different populations of *E. martini* have diverged in oocyte production (greater in the temperate population) as well as body size, suggesting that temperature could facilitate population differentiation (McKie *et al*., 2004). Indeed, tropical species often have higher thermal limits than congeneric temperate species (Chapman, 2013). For EPT species of Ecuadorian Andean and USA Rocky Mountains, Polato *et al*. (2018) showed that tropical and temperate mountain stream insects have diverged in thermal tolerance and dispersal capacity due to different seasonal temperature variations. Tropical species had narrower thermal breadths, less gene flow, higher population divergence, higher cryptic diversity, and higher speciation rates, rendering them especially vulnerable to rapid changes in thermal environments. Johansson, Quintela & Laurila (2016) showed that the genetic population structure of the Icelandic freshwater gastropod *Radix balthica* living in contrasting geothermal habitats was influenced by both geographic distance and water temperature. Genetic variation decreased with increasing temperature, suggesting that natural selection had led to reduced genetic diversity in warm geothermal springs due to higher thermal specialization. Herzog & Hadrys (2017) used a 20‐year data set on the genetic diversity of a population of *Orthetrum coerulescens* (Odonata) located in Crau (France) to identify a dramatic decline in genetic diversity caused by increased water temperatures mediated by the destruction of bank vegetation. At high altitudes, global warming is leading to the loss of glaciers, which promotes fragmentation, limits gene flow and leads to loss of genetic variation among populations of high‐altitude freshwater invertebrates. For example, populations of *Lednia tumana* (Plecoptera) in the Glacier National Park (Montana, USA) showed reduced gene and nucleotide diversity and increased genetic isolation in response to glacier retreat during 1997–2010 (Jordan *et al*., 2016). Genetic loss has been predicted in some European meltwater species of stoneflies, caddisflies and mayflies from predictions of mitochondrial DNA variability under different climate change scenarios. Intraspecific (cryptic) genetic loss is a significant concern and should be included when estimating the effects of global warming on biodiversity loss (Bálint *et al*., 2011). Temperature and genetic variability may interact affecting mortality: dramatic effects of high temperature (20 °C) were observed in a population of *Chironomus riparius* (Diptera) with poor genetic variability (Vogt *et al*., 2007).

## V. FUTURE DIRECTIONS

As shown in Fig. 7 there has been a shift in emphasis in research on the effects of temperature on aquatic macroinvertebrates from the 2000s, when studies at larger temporal (decades) and spatial (ecoregions, continents and global) scales began to be published. This increase is likely due to the availability of large databases and improved analytical tools. However, most studies on macroinvertebrate communities (now often related to rising water temperatures due to global warming) do not report measured water temperature, but instead use air temperature as a proxy (Li *et al*., 2013; Domish *et al*., 2011, 2013; Jourdan *et al*., 2018; Besacier *et al*., 2019; Haase *et al*., 2019). This approach may be limiting because it does not account for the high thermal heterogeneity of different water bodies nor their seasonal thermal variability. It is therefore essential to implement a monitoring network for water temperature, especially where there are few or no data. In recent years, several models have been developed that can effectively predict water temperature even at a regional scale and have proved useful both to reconstruct historical series and to enable temperature forecasts (Toffolon & Piccolroaz, 2015; Beaufort *et al*., 2016; Jackson *et al*., 2020). A deeper knowledge of the temperature conditions of aquatic ecosystems will allow evaluation of the effects of temperature alterations due to anthropogenic impacts, such as hydropower plants, deforestation, and thermal effluents. Detailed investigations of the temperature regime of freshwater bodies will provide the data necessary for a deep comprehension of aquatic processes related to temperature (Ducharne, 2008; Diamond *et al*., 2021) and thereby the effects of temperature on macroinvertebrates mediated by changes in oxygen availability, primary production, and organic matter availability (Verberk *et al*., 2021).

While we found many studies analysing different kinds of responses by macroinvertebrates both in the laboratory and in the field (Fig. 5), most of the literature regarding the effects of climate warming on macroinvertebrates concerns ecological responses related to community composition and taxa distribution (Fig. 6B) (Arai *et al*., 2015; Besacier *et al*., 2019). In recent years, studies have begun to investigate the effects of temperature on food‐web structure [e.g. trophic roles (Jonsson *et al*., 2015; Jourdan *et al*., 2018)] and trophic chain length (Glazier, 2012), but studies on the effects of climate warming on growth rates, phenology and fitness are relatively rare, despite a historical focus on these areas in laboratory studies (Fig. 6B). As noted by some authors (Forster *et al*., 2012; Tan *et al*., 2021; Verberk *et al*., 2021), warming induces earlier emergence and body size reduction in aquatic species, thus, the latitudinal temperature–size responses will influence the impacts of climate warming on primary production, community structure and food‐web dynamics.

Temperature affects physiology, phenology, and fitness, but these effects have been investigated almost exclusively at the population level, on target species studied in laboratory experiments (Fig. 6A,D). It will be important for future investigations to include these responses in field studies involving macroinvertebrate communities. Studying functional traits and ecosystem attributes (Cummins, 1974) can also include responses related to fitness, voltinism, trophic role and drift propensity that normally are not accounted for in structural taxonomy indices. Indeed, functional traits have already allowed a more comprehensive understanding of the effects of temperature changes on macroinvertebrate assemblages at large spatial and temporal scales (Hering *et al*., 2009; Poff *et al*., 2010; Pyne & Poff, 2017; Besacier *et al*., 2019). A trait‐based approach aiming to extend our knowledge about the thermal and ecological preferences of each taxon could be a promising way to understand and predict macroinvertebrate assemblage changes both in structure and functioning.

A further point of concern is the unbalanced number of studies among different continents (Figs. 6F and 9). We currently have very little information on the effects of water temperature on freshwater macroinvertebrates of 70% of our planet, with tropical, arid, and arctic climatic regions most unrepresented (Fig. 9). The focus of research is restricted mostly to European and North American macroinvertebrate communities, often living in water bodies profoundly impacted by direct human intervention (Dodds, Perkin & Gerken, 2013), and studies investigating the ecological effects of temperature have been carried out almost exclusively in Europe (Fig. 6F). This unbalanced distribution will inevitably lead to a somewhat distorted perception of macro‐ecological patterns related to temperature. Future work should target water bodies in Asia, Africa, South America, and Antarctica to allow us to obtain a more global perspective.

Genetic investigations of tropical and temperate macroinvertebrates have shown that taxon thermal preference varies across latitudes due to evolved thermal adaptations. Comparative studies demonstrated that tropical species generally have a narrower thermal range compared to temperate ones, occupy narrower ecological niches, are more specialized and therefore more vulnerable (Shah *et al*., 2017; Polato *et al*., 2018). However, information on the temperature ranges of tropical macroinvertebrate species is currently available only for about 60 species. According to Van Vliet *et al*. (2013) and Wanders *et al*. (2019), the water bodies most vulnerable to global warming are likely to be small, especially in temperate and arctic regions. It is, therefore, crucial to include more lentic ecosystems in thermal research given that lakes and ponds have been poorly investigated (Fig. 6A). They may be among the most vulnerable ecosystems to climate change because they are mainly in the boreal belt (Messager *et al*., 2016). Additionally, as reported by many authors (Bálint *et al*., 2011; Jordan *et al*., 2016; Shah *et al*., 2017; Polato *et al*., 2018; Birrell *et al*., 2020; Brighenti *et al*., 2021), mountain aquatic macroinvertebrates are likely to be the most threatened freshwater species due to their endemicity.

## VI. CONCLUSIONS

(1) Our review reveals that the effects of water temperature on macroinvertebrates are manifold with implications at different levels, from genes to communities, and involve multiple responses related to physiology, phenology, fitness, behaviour, community ecology and evolution. Despite substantial advances in thermal research in recent years, understanding how macroinvertebrate taxa and communities respond to different temperature conditions is far from complete.

(2) Temperature responses historically tested in laboratory studies, such as effects on body size, have not been assessed more widely at the community level. This will be necessary given their important macroecological implications.

(3) Studies involving gene expression have begun relatively recently. This promising avenue will provide an understanding of the physiological mechanisms underlying responses to temperature, and allow us to disentangle behavioural and evolutionary adaptation.

(4) Increasing temperatures driven by climate change strongly threaten stenothermal macroinvertebrates, especially in mountain waterbodies, but these are poorly investigated. When estimating biodiversity loss driven by warmer temperatures, both genetic and species loss should be considered.

(5) Tropical macroinvertebrates generally have a narrower thermal range and are more vulnerable to temperature changes; how they respond to changes in temperature should be examined in more detail.

(6) Our knowledge of macroinvertebrate–temperature relationships is based almost exclusively on studies carried out in the West, and in waterbodies greatly impacted by human activity; widening our perspective to include other regions will be important to enable a deeper understanding of the effects of climate change.

(7) Lakes and ponds are underrepresented in the published work and should be a focus of future studies because many are in the boreal belt that is warming faster that the global average.

(8) Extended spatiotemporal data sets often use air temperature as a proxy for water temperature and are derived almost exclusively for European rivers. Measuring water temperature data accurately will greatly improve our understanding of the effects of thermal heterogeneity on macroinvertebrate assemblage structure and the effects of temperature changes on aquatic ecosystem structure and functioning.

## Supporting information


**Fig. S1.** PRISMA flow diagram showing the different phases of article selection for the systematic review.
**Table S1.** Number of studies for each category of information extracted from the research publications (*N* = 218) included in the final database.

## Data Availability

Data are available at: https://doi.org/10.6084/m9.figshare.20584311.v1.
