# Supplementary material for: Effects of water temperature on freshwater macroinvertebrates: a systematic review
Source: Biol Rev Camb Philos Soc. 2022 Sep 29;98(1):191–221. doi: 10.1111/brv.12903 (PMC10088029; doi:10.1111/brv.12903)
Supplement: Supplementary file 1 — Fig. S1. PRISMA flow diagram showing the different phases of article selection for the systematic review. Table S1. Number of studies for each category of information extracted from the research publications (N = 218) included in the final database. [file BRV-98-191-s001.docx]

# Effects of water temperature on freshwater macroinvertebrates: a systematic review

Luca Bonacina^*^, Federica Fasano, Valeria Mezzanotte and Riccardo Fornaroli

*Department of Earth and Environmental Sciences (DISAT), University of Milano-Bicocca, Piazza della Scienza 1, 20126 Milan, Italy*

**Identification**

**Screening**

**Eligibility**

**Included**

Publications identified through database searching (*N* = 425)

Publications screened (*N* = 425)

Publications excluded, with reasons (*N* = 156)

Publications assessed for eligibility (*N* = 269)

Publications excluded, with reasons (*N* = 46)

Research articles included in quantitative synthesis (*N* = 218)

Publications included in qualitative synthesis (*N* = 223):

218 research articles and 5 reviews

**Fig. S1.** PRISMA flow diagram showing the different phases of article selection for the systematic review.

**Table S1.** Numbers of studies for each category of information extracted from the research publications (N = 218) included in the final database.

| **Category:** | **Ecosystem** | | **Study** | | **Continent** | | **Climatic region** | | **Spatial scale** | | **Temporal scale** | | **Sampling frequency** | | **Level of investigation** | |
| --- | --- | --- | --- | --- | --- | --- | --- | --- | --- | --- | --- | --- | --- | --- | --- | --- |
|  | channel | 8 | experimental | 194 | Africa | 5 | arid | 11 | catchment | 45 | days | 27 | annual | 7 | family | 15 |
|  | laboratory | 128 | theoretical | 24 | Antarctica | 2 | cold | 68 | continental | 8 | decades | 15 | daily | 57 | genus | 9 |
|  | lake | 11 |  |  | Asia | 10 | polar | 6 | ecoregion | 4 | months | 103 | hourly | 14 | order | 4 |
|  | pond | 5 |  |  | Central America | 2 | temperate | 139 | global | 4 | punctual | 14 | monthly | 52 | species | 191 |
|  | river | 74 |  |  | Europe | 100 | tropical | 13 | regional | 32 | years | 59 | punctual | 16 |  |  |
|  | spring | 9 |  |  | North America | 92 |  |  | site-specific | 109 |  |  | seasonal | 4 |  |  |
|  |  |  |  |  | Oceania | 16 |  |  |  |  |  |  | subhourly | 7 |  |  |
|  |  |  |  |  | South America | 11 |  |  |  |  |  |  | weekly | 36 |  |  |
| Sum |  | 235 |  | 218 |  | 238 |  | 237 |  | 202 |  | 218 |  | 193 |  | 219 |
|  | not found | 10 | not found | 0 | not found | 7 | not found | 8 | not found | 16 | not found | 11 | not found | 41 | not found | 3 |
| **Total** |  | **245** |  | **218** |  | **245** |  | **245** |  | **218** |  | **229** |  | **234** |  | **222** |
|  |  |  |  |  |  |  |  |  |  |  |  |  |  |  |  |  |
| **Category:** | **Ecological unit** | | **Organism** | | | | **Temperature conditions** | | **Other stress** | | **Effects** | | **Responses** | | |  |
|  | community | 59 | Arachnida | | Trombidiformes | 1 | climate warming | 20 | flow | 6 | behaviour | 24 | drift | | | 7 |
|  | gene | 8 | Bivalvia | | Cardiida | 1 | constant thermal regime | 7 | food availability | 19 |  |  | feeding | | | 7 |
|  | population | 151 |  | | Sphaeriida | 1 | high temperature | 16 | habitat | 3 |  |  | migration | | | 4 |
|  |  |  |  | | Unionida | 1 | natural thermal regime | 74 | humidity | 3 |  |  | predation | | | 6 |
|  |  |  |  | | Veneroida | 1 | temperature range | 120 | nutrient concentration | 3 | ecological | 132 | community structure and trophic role | | | 3 |
|  |  |  | Clitellata | | Arhynchobdellida | 3 | temperature variation | 8 | other | 7 |  |  | density | | | 34 |
|  |  |  |  | | Haplotaxida | 4 |  |  | oxygen availability | 6 |  |  | distribution | | | 25 |
|  |  |  |  | | Lumbriculida | 1 |  |  | photoperiod | 9 |  |  | food-chain length | | | 1 |
|  |  |  |  | | Opisthopora | 2 |  |  | pollution | 3 |  |  | richness | | | 31 |
|  |  |  | Gastropoda | | Littorinimorpha | 5 |  |  | predation | 7 |  |  | secondary production | | | 7 |
|  |  |  |  | | Neotaenioglossa | 1 |  |  |  |  |  |  | taxonomic composition | | | 31 |
|  |  |  |  | | Pulmonata | 2 |  |  |  |  | evolutionary | 4 | genetic diversity | | | 4 |
|  |  |  | Insecta | | Coleoptera | 17 |  |  |  |  | fitness | 70 | fecundity and hatching success | | | 47 |
|  |  |  |  | | Diptera | 57 |  |  |  |  |  |  | larval recruitment | | | 23 |
|  |  |  |  | | Ephemeroptera | 80 |  |  |  |  | phenological | 132 | colour | | | 4 |
|  |  |  |  | | Mecoptera | 1 |  |  |  |  |  |  | time and length of emergence | | | 49 |
|  |  |  |  | | Neuroptera | 5 |  |  |  |  |  |  | time and length of hatching | | | 39 |
|  |  |  |  | | Odonata | 61 |  |  |  |  |  |  | total time of development | | | 29 |
|  |  |  |  | | Plecoptera | 55 |  |  |  |  |  |  | voltinism | | | 11 |
|  |  |  |  | | Rhynchota | 5 |  |  |  |  | physiological | 163 | assimilation/excretion | | | 16 |
|  |  |  |  | | Trichoptera | 57 |  |  |  |  | and metabolic |  | body size and growth rate | | | 85 |
|  |  |  | Malacostraca | | Amphipoda | 11 |  |  |  |  |  |  | gene expression | | | 9 |
|  |  |  |  | | Decapoda | 3 |  |  |  |  |  |  | osmoregulation ability | | | 1 |
|  |  |  |  | | Harpacticoida | 1 |  |  |  |  |  |  | respiration | | | 8 |
|  |  |  |  | | Isopoda | 8 |  |  |  |  |  |  | size at emergence | | | 19 |
|  |  |  |  | | Mysida | 1 |  |  |  |  |  |  | thermal limits | | | 25 |
|  |  |  | Maxillopoda | | Cyclopoida | 2 |  |  |  |  |  |  |  | | |  |
|  |  |  | Rhabditophora | | Tricladida | 3 |  |  |  |  |  |  |  | | |  |
| Sum |  | 218 |  | | | 390 |  | 245 |  | 66 |  | 525 |  | | | 525 |
|  | not found | 0 |  | | not found | 3 | not found | 0 | not found | 0 | not found | 0 | not found | | | 0 |
| **Total** |  | **218** |  |  |  | **393** |  | **245** |  | **66** |  | **525** |  | | | **525** |
